# Omega-3 Fatty Acid Supplementation on Post-Exercise Inflammation, Muscle Damage, Oxidative Response, and Sports Performance in Physically Healthy Adults—A Systematic Review of Randomized Controlled Trials

**DOI:** 10.3390/nu16132044

**Published:** 2024-06-27

**Authors:** Diego Fernández-Lázaro, Soledad Arribalzaga, Eduardo Gutiérrez-Abejón, Mohammad Ali Azarbayjani, Juan Mielgo-Ayuso, Enrique Roche

**Affiliations:** 1 Department of Cellular Biology, Genetic, Histology and Pharmacology, Faculty of Health Sciences, University of Valladolid, Campus de Soria, 42003 Soria, Spain; 2 Neurobiology Research Group, Faculty of Medicine, University of Valladolid, 47005 Valladolid, Spain; 3 “Nutrition for Sport and Exercise” Working Group, Spanish Nutrition Society (SEÑ), 28010 Madrid, Spain; jfmielgo@ubu.es (J.M.-A.); eroche@umh.es (E.R.); 4 Faculty of Sport Sciences, European University of Madrid, Villaviciosa de Odón, 28670 Madrid, Spain; 5 Pharmacological Big Data Laboratory, Department of Cell Biology, Genetics, Histology and Pharmacology, Faculty of Medicine, University of Valladolid, 47005 Valladolid, Spain; eduardo.gutierrez.abejon@uva.es; 6 Valladolid Este Primary Care Department, 47005 Valladolid, Spain; 7 Pharmacy Directorate, Castilla y León Health Council, 47005 Valladolid, Spain; 8 Department of Exercise Physiology, Central Tehran Branch, Islamic Azad University, Tehran 14778-93855, Iran; m_azarbayjani@iauctb.ac.ir; 9 Department of Health Sciences, Faculty of Health Sciences, University of Burgos, 09001 Burgos, Spain; 10 Department of Applied Biology-Nutrition, Institute of Bioengineering, University Miguel Hernández, 03202 Elche, Spain; 11 Alicante Institute for Health and Biomedical Research (ISABIAL), 03010 Alicante, Spain; 12 CIBER Fisiopatología de la Obesidad y Nutrición (CIBEROBN), Instituto de Salud Carlos III (ISCIII), 28029 Madrid, Spain

**Keywords:** omega-3 fatty acids, inflammation, muscle damage, oxidative response, sports performance, sports nutrition, supplementation

## Abstract

Omega-3 is a family of n-3 polyunsaturated fatty acids (PUFAs), which have been used to treat a wide variety of chronic diseases, due mainly to their antioxidant and anti-inflammatory properties, among others. In this context, omega-3 could be post-exercise recovery agent and sports supplement that could improve performance by preserving and promoting skeletal muscle mass and strength. No conclusive evidence, however, exists about the potential effects of omega-3 on post-exercise biomarkers and sports performance in physically healthy adults. Based on the PRISMA in Exercise, Rehabilitation, Sports Medicine, and Sports Science (PERSiST) guidelines, we systematically reviewed studies indexed in Web of Science, Scopus, and Medline to assess the effects of omega-3 on post-exercise inflammation, muscle damage, oxidant response, and sports performance in physically healthy adults. The search was performed on original articles published in the last 10 years up to 5 May 2024, with a controlled trial design in which omega-3 supplementation was compared with a control group. Among 14,971 records identified in the search, 13 studies met the selection criteria. The duration of the interventions ranged from 1 day to 26 weeks of supplementation and the doses used were heterogeneous. Creatine kinase (CK) and lactate dehydrogenase (LDH) were significantly higher (*p* < 0.05) in the control group in 3 of the 4 studies where these markers were analyzed. C-reactive protein (CRP) was significantly higher (*p* < 0.05) in the control group of 2 of the 13 studies where this marker was analyzed. The delayed onset muscle soreness (DOMS) gave mixed results. Interleukin 6 (IL-6) showed improvements with supplementation, but tumor necrosis factor-α (TNF-α) displayed no differences. The consumption of n-3 PUFAs improved some indicators of oxidative stress such as reduced glutathione (GSH)/oxidized glutathione (GSSG) ratio. Additional evidence is needed to establish clear recommendations regarding the dose and length of n-3 PUFA supplements. These may benefit the post-exercise inflammatory response, mitigate muscle damage, and decrease oxidative stress caused by exercise. However, studies did not evaluate omega-3 status at baseline or following supplementation and therefore the observations must be treated with caution

## 1. Introduction

Oxidative stress is defined as a disturbance in the balance between free radicals and antioxidants in biological systems, in favor of the first [1]. Therefore, oxidative stress is characterized by an increase in free radicals and pro-oxidant compounds, along with a decrease in antioxidants, leading to oxidative damage to cellular components such as lipids, proteins, and deoxyribonucleic acid (DNA) [2]. Additionally, there is a link between oxidative stress, inflammation, and muscle damage [3], because the former processes can trigger the latter. In this line, inflammatory cells can promote oxidative stress by releasing enzymes (neutral proteases, elastase, collagenase, acid hydrolases), reactive species (superoxide, hydrogen peroxide, hydroxyl radical, hypochlorous acid, nitric oxide), and extracellular messengers (cytokines and interleukins). Altogether, these compounds can exacerbate inflammation and muscle damage [4]. In this context, it is important to note that during physical activity, muscles produce free radicals [5]. The production of Reactive Oxygen Species (ROS) during muscle contraction occurs through various mechanisms, including the activation of endothelial xanthine oxidase, increased release and autooxidation of catecholamines, electron leakage in the mitochondrial electron transport chain, and the previously mentioned inflammatory response [2,3]. During exercise and particularly in demanding routines, the production of ROS often surpasses muscle antioxidant capacity. If this imbalance persists, it can reduce force production, contributing to the onset of acute fatigue, decreased sports performance, increased muscle damage, and even health problems [6].

To combat this unbalance, sports nutrition must be used to cover the greater energy and nutrient requirements that demanding physical activity entails to improve health, sports performance, and recovery [7]. Research on sports supplements has focused on those that play a beneficial role in recovery and reduce the risk of injury or illness [8]. Omega-3 polyunsaturated fatty acids (n-3 PUFA), part of the polyunsaturated fatty acid family, are among these supplements. In addition, omega-3 fatty acids, particularly EPA and DHA, play a crucial role in human physiology by contributing to cell membrane fluidity, reducing inflammation, and supporting cardiovascular health. They are essential for brain function and development, and their anti-inflammatory properties can help manage chronic diseases and improve overall health. Since n-3 PUFA cannot be produced by the human body, it is crucial to obtain them through diet or supplementation. There are three primary omega-3 fatty acids: alpha-linolenic acid (ALA, 18:3 n-3), eicosapentaenoic acid (EPA, 20:5 n-3), and docosahexaenoic acid (DHA, 22:6 n-3). ALA is found in plant-based sources such as flaxseed, chia seeds, and walnuts, while EPA and DHA are primarily found in seafood and fatty fish like salmon, tuna, and mackerel [9]. Although ALA can be converted into EPA and DHA in the organism, this conversion process is limited and suboptimal, making it preferable to consume EPA and DHA directly from foods or supplements [10]. Nevertheless, ALA is the only essential omega-3 fatty acid due to the body’s inability to synthesize it; however, the low conversion efficiency suggests that direct intake of EPA and DHA might be necessary to meet physiological needs, especially for individuals with specific health conditions or dietary restrictions. Direct dietary sources (like oily fish) or supplements (the present review) may be needed to ensure adequate levels, supporting overall health and addressing specific needs like cardiovascular health, cognitive function, and inflammation. Therefore, it is important to highlight the potential gap in omega-3 intake when relying solely on ALA. Therefore, the role of EPA and DHA in maintaining optimal health might be considered. In this context, the amount of oily fish required to achieve typical supplement doses of 18–3.5 g of n-3 PUFA (EPA and DHA combined) depends on the type of fish, being for some common oily fish of 1.5–2.5 g (salmon), 1.5–2 g (herring), 1–1.5 g (mackerel, sardines) and 0.5–1.6 g (tuna) of omega-3 content per 100 g. Therefore, supplementation (the topic of the present review) allows athletes to ensure specific amounts of these fatty acids in a more convenient manner.

Several studies have demonstrated the benefits of n-3 PUFA consumption for athletes. These benefits include reduced fatigue [11], decreased proinflammatory cytokine production, particularly documented in athletes with asthma [12], reduced production of eicosanoids [13], increased muscle mass and strength [14], positive impact on recovery processes [15], and improved adaptation to training [16,17]. Additionally, n-3 PUFAs can reduce the activation of the NF-κB pathway by preventing degradation and translocation of this transcription factor to the nucleus [18]. This leads to lower circulating concentrations of tumor necrosis factor-α (TNF-α), which acts as a negative feedback mechanism on NF-κB activation [19].

In addition, the benefits of n-3 PUFA on neural function [20] and exercise adaptations [21] can be attributed to several mechanisms, including improved nerve conduction [22], increased sensitivity to acetylcholine [23], enhanced membrane fluidity, and reduced post-exercise inflammation [24]. Furthermore, the positive effect of this supplement on muscle protein synthesis is linked to the fact that n-3 PUFAs sensitize skeletal muscle to anabolic stimuli, through resistance exercises accompanied or not with protein intake [25]. Furthermore, n-3 PUFA consumption enhances the activation of the rapamycin complex pathway (mTORC), a key player in muscle protein synthesis [26]. Finally, n-3 PUFA supplementation has shown benefits when combined with specific physical tests. For example, a study involving omega-3 supplementation and the Wingate test together with a 250 kJ time trial, followed by measurements of maximal voluntary isometric contraction, resulted in an increase in quadriceps peak force, improved muscle activation, and reduced lag time between muscle electrical activity and the onset of contraction [27].

Therefore, we aimed to systematically review the current evidence on the effects of omega-3 on health and sports biomarkers and determine whether the supplementation of omega-3 (EPA/DHA) improves inflammation markers, muscle damage, oxidant response, and sports performance in physically healthy adults.

## 2. Materials and Methods

### 2.1. Search Strategy

This systematic review was carried out following the guidelines of the Preferred Reporting Items for Systematic Review and Meta-Analyses (PRISMA^®^) in Exercise, Rehabilitation, Sports Medicine and Sports Science (PERSiST) [28] and the PICOS [29] model to define inclusion criteria: P (population): “physically healthy adults”, I (Intervention): “Omega-3 supplementation”, C (Comparison): “same conditions with placebo, sham treatment or control group”, O (Outcomes): “inflammation markers, muscle damage, oxidant response, and sports performance”, S (study): “randomized controlled trials”.

A structured search was conducted in Medline (Pubmed), Scopus, and Web of Science (WOS) for articles published in the last 10 years until 5 May 2024, restricted to English language articles, and based on PERSiST [28] guidelines. The search strategy included terms related to omega-3 and the outcomes as well as a combination of them with Medical Subject Headings (MeSH) index and Boolean operators: (“omega-3” OR “omega-3 supplementation” OR “Polyunsaturated fatty acids”) AND (“muscle recovery”), AND (“athletic performance” OR “improved athletic performance”) AND (“exercise-induced muscle damage” OR “muscle soreness” OR “muscle damage”) AND (“eccentric exercise”) AND (“inflammation” OR “oxidative stress”) AND (“benefits”) (Appendix A).

Relevant articles were also obtained using this equation by applying the snowball strategy. This approach allows all titles and abstracts from the search to be cross-referenced to identify duplicates and any potentially missing studies. Titles and abstracts were selected for further review of the full text. The search for published studies was independently performed by 2 authors (D.F.-L. and S.A.) and disagreements about records were resolved by a third reviewer (E.R.).

### 2.2. Selection Criteria

The following selection criteria were applied in choosing studies for the articles obtained from the search according to the above mentioned inclusion criteria (Section 2.1): (i) physically healthy adults ≥ 18 age, without chronic conditions; (ii) studies that evaluated the effects of omega-3 supplementation alone, excluding drugs formulas and any combination with other supplements (mono-supplementation); (iii) randomized controlled trials (excluding editorials, reviews, notes, and any others from non-original studies); (iv) studies that evaluated as outcomes (primary, secondary, or safety) any of the inflammation markers, muscle damage, oxidant response, and sports performance; (v) studies with clear information on dosage and duration of omega-3 supplementation; (vi) studies with a methodological quality score ≥ 10 according to the McMaster University Occupational Therapy Evidence-Based [30]; (vii) clinical trials or randomized clinical trials with a score ≥ 8 on the Physiotherapy Evidence Database (PEDro) scale [31]; and (viii) studies published in English. We excluded all records that did not meet these criteria.

### 2.3. Study Selection

Once the inclusion/exclusion and selection criteria (Section 2.1 and Section 2.2) have been applied to each study, data on study source including authors and year of publication, study design, supplement administration (dose and schedule), sample size, participant characteristics (age, height, weight, fat percentage, body mass index, and sex), and outcomes of the interventions were mined independently by two authors (D.F.-L. and S.A.) using a spreadsheet 4.1.2 (Microsoft Inc, Seattle, WA, USA). Subsequently, disagreements were resolved through discussion until a consensus was reached or by a third reviewer (E.R.) when a consensus was not reached.

### 2.4. Quality Assessment

We used the modified critical review form for quantitative studies developed by McMaster University Occupational Therapy Evidence-Based Practice Research Group (McMaster) as a critical appraisal tool [30]. Additionally, the Physiotherapy Evidence Database (PEDro) scale [31] was used to assess the methodological quality of quantitative evidence. Two authors independently evaluated the methodological quality (D.F.-L. and J.M.-A.), resolving disagreements through third-party evaluation (E.G.-A).

### 2.5. Risk-of-Bias Assessment

Furthermore, the included studies were assessed using the Cochrane risk of bias (RoB) [32]. The risk of bias was evaluated by two authors independently (D.F.-L. and S.A.), resolving disagreements through third-party evaluation (E.G.-A.).

### 2.6. Data Extraction

Once the inclusion and exclusion criteria were applied, relevant information was gathered from the chosen studies. Two reviewers (D.F.-L. and E.R.) examined and synthesized data from all selected studies into a comprehensive table using a standardized data extraction. A third reviewer (S.A.) resolved all inter-reviewer disagreements. The extracted data included the name of the primary author, publication year, country of origin, study design, sample size, participant characteristics (such as sex, age, level of physical activity, and health status), details of the intervention (including the daily dosage of omega-3 supplementation and the periods in which the supplementation was taken), variables analyzed, and the corresponding results.

## 3. Results

### 3.1. Study Selection

**Figure 1:**
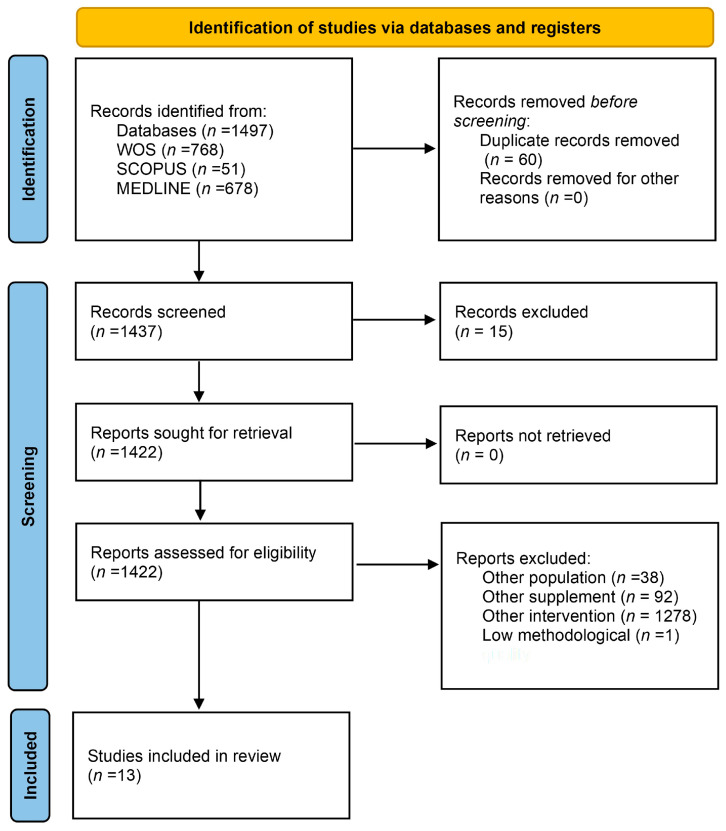
Flow diagram depicting the identification and selection processes of relevant studies according to PRISMA guidelines.

A total of 1497 studies were identified from WOS (*n* = 768), SCOPUS (*n* = 51), and Medline (*n* = 678). After the exclusion of 60 duplicates, a total of 1437 articles identified in databases were examined. After evaluation of the title and abstract, 1422 articles were considered as potential documents. After a review of the full text and evaluation of potential records from databases as well as other sources, 13 studies [16,17,33,34,35,36,37,38,39,40,41,42,43] were included in the systematic review (Figure 1).

### 3.2. Quality Assessment

Five studies [16,17,35,37,38] were considered “*excellent quality*” and 8 [33,34,36,39,40,41,42,43] as “*very good quality*” according to McMaster [30] (Table 1). Also, according to the PEDro scale [31], 10 studies [16,17,34,35,36,37,38,39,40,42,44] had a rating of “*excellent quality*” and 3 studies [33,41,43] of “*good quality*” (Table 2).

### 3.3. Risk-of-Bias Assessment

**Figure 2:**
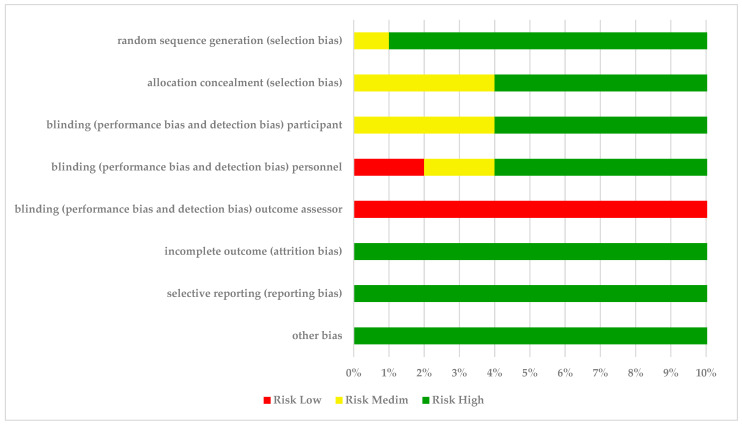
The most common problems found in the risk of bias in selected studies.

Table 3 shows the results of the RoB assessment tool [32] applied to the studies in this review. Figure 2 presents a summary of the author’s judgments on each RoB item for each included study. Regarding selection bias, 12 studies [17,33,34,35,36,37,38,39,40,41,42,43] reported an adequate method to generate the randomization sequence of the participants and were judged as low RoB. Additionally, 9 studies [17,33,34,35,36,38,39,40,41] detailed the allocation concealment process and were also considered with low RoB. Blinding of participants was judged as a medium RoB in [33,39,41,43] for blinding personnel, a medium RoB in [33,39] and high RoB in [16,41]. Regarding the blinding of outcome assessors’ item, one study [40] had a low RoB and all the other studies were classified as high RoB. For the final three items (attrition bias, reporting bias, and other bias) all studies were evaluated as having low RoB [16,17,33,34,35,36,37,38,39,40,41,42,43].

### 3.4. Outcome Evaluation

The sample and supplementation characteristics are in Table 4. Table 5 shows the records included in the systematic review of the effect of omega-3 supplementation on post-exercise inflammation markers, muscle damage, oxidative response, and sports performance in physically healthy adults.

#### 3.4.1. Characteristics of the Sample

Thirteen registries were analyzed [16,17,33,34,35,36,37,38,39,40,41,42,43], with a study population that varied between 18 [38] and 69 [16] participants: 5 of them were performed with men and women [16,35,39,41,43], 1 study exclusively in women [38], and 7 studies exclusively in men [17,33,34,36,37,40,42], with a total of 420 participants (57 withdrawals; 74 women and 404 men). One of the inclusion criteria of the studies is that they had to perform the intervention in physically healthy adults, including competitive amateur or trained college students. Considering the type of exercise performed during the intervention, selected articles can be classified into three groups. In the first group, participants performed endurance exercises [36,43], strength exercises in the second group [16,17,34,35,37,38,39,40,41], and a combination of both in the third group [33,35]. The endurance intervention was performed by running [43] or cycling [36] to exhaustion or structured endurance training (31 km per week) [33] and no details of the exercises [41,42]. The strength exercises were performed in the upper body [16,17,34], lower body [35,40,45], specifically through vertical jumps [35,40], or no details of exercises were provided [37].

#### 3.4.2. Omega-3 Supplementation

The total supplementation dose was recorded in the group that ingested the lowest dose of 250 mg/day (150 mg EPA + 100 mg DHA) [40] in one of the experimental groups with a maximum of 31 g/day ALA (0.43 g ALA kg/body mass (BM) for the average [43]). The remaining doses ranged from 1.386 g [37] to 3.6 g/day [38]. Considering the duration of the intervention, the shortest was one day [40] and a maximum of 26 weeks [42]. Regarding the mode of administration of the supplements, some studies used capsules [16,17,34,35,37,39,40,41], gels [36,42], flavored water with seed [43], or it was not specified [33,38]. In two studies, the intake was performed in relation to exercise—before [43] or after [40]—or after meals [17,34]. Ten studies did not provide information regarding schedules [16,33,35,36,37,38,39,41,42,46]. No other harmful symptoms or adverse effects were reported by omega-3 supplementation [16,35,39].

#### 3.4.3. Inflammatory Markers

IL-6 was analyzed in seven records [33,34,37,40,41,42,43] where five of them showed no significant changes (*p* > 0.05) [33,34,40,42,43] and in two [37,41] IL-6 decreased significantly (*p* < 0.05) when comparing pre-and post-intervention in the supplemented group. No significant (*p* > 0.05) changes were observed in IL-6 when the supplemented group was compared with the control group (CG) in five studies [33,34,40,42,43]. In two studies [37,41], IL-6 was significantly higher (*p* < 0.05) in CG. Interleukin-1β (IL-1β) was analyzed in one study [37], with no significant changes between pre- and post-exercise values as well as comparing CG with the supplemented group (*p* < 0.05). Interleukin-2 (IL-2) [46], interleukin-8 (IL-8), and interleukin-10 (IL-10) were also evaluated [43] and no significant differences between groups were found.

TNF-α was analyzed in four investigations [37,41,42,43], in none of them the group supplemented with n-3 PUFA presented significant changes (*p* > 0.05) comparing pre- and post-exercise. In the same reports, differences between supplemented and CG were not significant (*p* > 0.05) in two studies [37,42]. In the study by Lee et al., [41] the CG presented higher levels of TNF-α.

C-reactive protein (CRP) was evaluated in two articles [37,41] and significant differences (*p* > 0.05) between groups were found. In both the study by Lee et al. [41] and Barquilha et al. [37] the groups were compared, resulting in significantly higher levels (*p* < 0.05) of CRP in the CG.

#### 3.4.4. Muscle Damage

Muscle damage was assessed with different variables, including creatine kinase (CK) and lactate dehydrogenase (LDH) presence in serum [47]. These biomarkers were analyzed in six studies [16,17,34,37,40,41]. One study [40] showed a significant increase (*p* < 0.05) in the first 24 h in both groups compared to the initial value. On the contrary, one study [37] presented significantly (*p* < 0.05) lower values after supplementation with DHA + EPA. On the other hand, the studies by Tsuchiya et al. [17] and Lembke et al. [16] presented no significant (*p* > 0.05) changes in serum CK levels after exercise compared to previous values in any of the groups and in any of the measurements over time [16]. Similar results were presented in the other study by Tsuchiya et al. [34] where no significant differences were observed after exercise compared to pre-exercise values in the EPA and DHA group. Serum CK levels were significantly higher in the CG than in the EPA and DHA group 3 days after exercise. One study [35] analyzed different amounts of omega-3 and in comparison with CG, CK tended (*p* = 0.055) to be lower at 72 h for the group that consumed the highest dose (6 g) of supplement. This study seems to correlate a greater amount of supplement with a lower value for CK. This was confirmed through determinations of CK over intervention time; meanwhile, CK increased in CG. When comparing the supplemented group with the CG, three studies [34,35,37] evidenced that the values of circulating CK were significantly higher (*p* < 0.05) in the CG. This was observed in VanDusseldorp et al. [35] after 72 h.

LDH values were analyzed in two studies [35,37]. In Barquilha et al. [37], the CG and the omega-3-consuming group reported an increase in pre-exercise activity, but in the post-exercise period, these values were attenuated in the CG and reduced in the supplementation group. The study by VanDusseldorp et al. [35] used three different doses of omega-3. When the lower dose (2 g) was provided, the circulating levels of LDH were higher compared to the higher supplementation dose (6 g) at 4, 24, 46, and 72 h post-exercise. In two studies [35,37], the LDH values in CG were significantly (*p* < 0.05) higher than in the supplemented group. In the case of VanDusseldorp et al. [35] the difference was significant (*p* > 0.05) after 72 h in CG.

Only one study analyzed myoglobin circulating levels [17]. Serum levels increased in the CG, meanwhile no significant changes in myoglobin levels were observed in the omega-3 group. Another indicator of damage is cortisol, being measured in one study [43]. Although cortisol circulating values were elevated after exercise, no differences between groups were reported.

Delayed Onset Muscle Soreness (DOMS) was analyzed in five studies [16,17,34,35,40]. Jakeman et al. [40] showed significant increases (*p* < 0.05) in the omega-3 group immediately at the end of exercise, decreasing subsequently. On the contrary, in one study [34], participants displayed a significant increase (*p* < 0.05) of DOMS in the first 4 days. VanDusseldorp et al. [35] made comparisons with different doses of the supplement. The effect of dosage on the interaction between treatment and time on perceived pain was studied. Pain tended to be elevated for the CG and the lower dose (2 g) in the supplemented group. In addition, the pain scores of the lower dose groups did not increase significantly until 24 h, remaining elevated at 28 h. Conversely, the higher dose group (6 g) reported lower pain scores compared to the 2- and 4-g groups. Two studies [34,40] showed no significant difference (*p* > 0.05) between supplemented compared to CG.

#### 3.4.5. Oxidant Response

Oxidative stress was evaluated in one study [37]. Barquilha et al. [37] analyzed the Trolox equivalent antioxidant capacity (TEAC), reduced (GSH), and oxidized (GSSG) glutathione. It was found that supplementation did not change TEAC values 24 h after strength exercise compared to CG. The values for GSH increased and those for GSSG decreased. Consequently, the GSH/GSSH ratio increased with omega-3 supplementation.

#### 3.4.6. Sports Performance

Three studies [17,34,35] evaluated the maximal voluntary isometric contractions (MVIC), presenting a significant decrease (*p* < 0.05) at the end. No significant changes (*p* > 0.05) were observed when compared to CG in [34].

In performance, the squat with a jump and jump with countermovement [40] presented a significant effect, which decreased significantly (*p* < 0.05) at one hour and was maintained until 96 h. The performance of the squat with the jump was significantly better when the amount of supplementation increased. In the case of vertical jump performance [35], this recovered pre-exercise values within 1 h for the group supplemented with the highest dose (6 g) while it remained low for the rest of the groups until 48 h (CG, 2 g, and 4 g of supplement).

In one study [40], the isokinetic strength evaluated did not show differences concerning the CG (*p* > 0.05) and decreased as time passed. Muscle strength determined through one repetition maximum (1RM) was analyzed in different exercises [41], in which a significant increase (*p* < 0.05) was observed in the supplemented group compared to the CG. In this line, Heileson et al. [39] evaluated 1RM in squat and bench. The supplemented group increased absolute 1RM-Bench and tended to increase in absolute 1RM-Squat, compared to CG. The study [38] analyzed 1RM and maximum voluntary contraction (MVC) in a trained leg. The results showed a progressive increase in 1RM in the trained leg for the experimental and CGs but no increases in the MVC of the trained leg between groups or throughout the protocol. Regarding the eccentric speed analyzed in one study [35], a significant increase (*p* < 0.05) was observed at the end of the exercise in the supplemented group.

Range of motion (ROM) was evaluated in two studies [17,34]. In one of them [17], there was no significant decrease in elbow ROM to pre-exercise values in the omega-3 group, but a significant decrease was observed in CG immediately after exercise lasting below initial levels for 1–3 days. In the study by the same author but 5 years later [34], a similar pattern was observed in the CG, and the omega-3 group decreased immediately after exercise, lasting below initial levels only for 1 day. However, the supplemented group returned to pre-exercise values on day 2 after exercise [34]. In both studies [17,34], the elbow ROM in the omega-3 group was significantly higher than in the CG immediately after exercise and 3 days later.

Regarding muscle echo intensity through ultrasound pictures, in the study of Tsuchiya et al. [34] no significant increase (*p* > 0.05) was observed comparing both groups (supplemented vs. CG). Nevertheless, the M-wave latency investigated was significantly higher (*p* < 0.05) in the supplemented group.

## 4. Discussion

The purpose of this systematic review was to critically evaluate the effects of omega-3 supplementation on post-exercise inflammatory biomarkers, oxidative response, muscle damage, and sports performance in physically active healthy adults. A total of 13 studies met the inclusion criteria. Participants supplemented with omega-3 presented improvements in attenuating biomarkers of muscle damage, such as CK and LDH. Inflammatory biomarkers showed positive effects on eccentric exercise protocols by decreasing concentrations of IL-6 and TNF-α. However, there was no clear evidence of the beneficial effects of omega-3 supplementation on muscle function and performance. Also, supplementation of 2400 mg/day of omega-3 fatty acids (EPA/DHA) for 4.5 weeks appears to be an effective dose.

### 4.1. Omega-3 Supplementation

All studies were conducted in physically healthy adults. The amounts used in the selected studies ranged from 250 mg [40] to 6 g per day [35] for EPA/DHA. Only one study used ALA with a dose of 0.43 g/kg body mass [43]. The duration of supplementation ranged from acute [43] to 26 weeks [42]. The consumption of 2400 mg/day of omega-3 fatty acids for 4.5 weeks [34] was effective when compared to other studies included in this systematic review using lower doses [33,35,36,37,41] or higher doses [16,17,35,38,40,42,43] and/or shorter [16,36,40,43] or larger time periods [17,33,35,37,38,39,41,42]. Particularly, the study conducted by Tinsley et al. [15] administered high doses (3.6 g of EPA/DHA) in short supplementation periods, such as 2 weeks.

A single study [35] examined the impact of three different doses: 2, 4, and 6 g/day with the same EPA/DHA ratio. The higher intake is more effective in delaying perceived muscle soreness at the end of the knee extensor strength series and on vertical jump performance.

In the study conducted by Kyriakidou et al. [46], 30 male athletes found that seven weeks of supplementation with 3 g per day reduced muscle soreness after eccentric exercise, without an effect on muscle function and post-exercise recovery at the end of exercise. Similar results were found in another study [48] that used the same daily amount but with a duration of 4 weeks.

On the other hand, one study [43] showed no clear results on the benefits of omega-3 fatty acids, particularly ALA, in physically active people during two intakes separated by two weeks. However, the studies that carried out interventions with different durations from one day [40] to 26 [42] weeks reported changes when EPA/DHA were supplemented. Some of them [16,17,34,35,37,40,41] informed that EPA/DHA consumption attenuated some of the markers of muscle damage and certain aspects of muscle soreness. Positive effects were documented as well in studies where the protocol involved eccentric forearm extensions [16], elbow flexors [17,34], or leg exercises [38,40,41] with supplementation periods ranging from one day [40] to 12 weeks [41], and with varying doses from 1 g per 10 kg of body weight [40] to 2.1 g/d EPA + 0.78 g/d DHA [41].

Some of the side effects derived from omega-3 supplementation are belching, heartburn or stomach pain, vomiting, constipation, diarrhea, nausea, and changes in the sense of taste [49]. However, three studies reported that omega-3 supplementation did not cause any harm or side effects [16,35,39]. However, no blood or tissue measurements were obtained from any of the studies reported [16,17,33,34,35,36,37,38,39,40,41,42,43], so the ability to confirm the change in omega-3 status is not possible in the current manuscript.

### 4.2. Inflammatory Markers

As mentioned before, the anti-inflammatory effect of EPA/DHA is due to the alteration of the inflammatory response through the production of specific lipid messengers [50]. The inflammatory process is characterized by the increase of prostaglandins, cytokines such as IL-6, IL-2, and TNF-α, CRP, and ROS. In addition, ROS produces peroxidation of phospholipid membranes, and damages in DNA and intracellular proteins [2].

EPA and DHA function as inhibitors of the endogenous synthesis of proinflammatory arachidonic acid, competing as substrates for enzymes during eicosanoid production [51]. Eicosanoids include prostaglandins, leukotrienes, and thromboxanes, key lipidic messengers in the inflammatory response [50]. Nevertheless, this response depends on the type, intensity, and duration of exercise, as well as the training level of the subject [52]. EPA/DHA alters the fatty acid composition of the phospholipid cell membrane, disrupting specialized regions with high concentrations of lipids such as cholesterol (lipid rafts), and inhibiting NF-κβ. These modifications favor decreased inflammatory gene expression and anti-inflammatory factor activation [53].

TNF-α is a cytokine that acts in the acute phase of inflammation. Main functions include increased synthesis of acute phase proteins produced in the liver such as CRP, as well as activation of monocytes and macrophages. Therefore, TNF-α promotes the inflammatory response. This is achieved by stimulating the expression of adhesion molecules on vascular endothelial cells, allowing the migration of inflammatory cells such as neutrophils and monocytes [54]. In this review, changes in TNF-α were not significant between groups [17,43], so the benefits of omega-3 supplements in this regard cannot be concluded. As an exception, Bloomer et al. [55] supplemented 14 men for 6 weeks with 2224 mg EPA and 2208 mg DHA, observing decreased levels of CRP and TNF-α compared to CG. However, no differences in oxidative stress markers were observed.

IL-6 is the main interleukin related to exercise, increasing after physical activity execution [37]. Omega-3 supplements can inhibit the activation of the NF-κβ pathway, which is responsible for activating inflammatory interleukins. Thus, when NF-κβ is inhibited, the production of cytokines such as IL-6, IL-2, and IL-8 is reduced. Aside from the inflammatory role, TNF-α and IL-6 participate in post-damage muscle regeneration by enhancing myoblast proliferation and inhibiting differentiation. This is achieved by decreasing the expression of the transcriptional regulatory factors myod and mygenin (regulators of myogenesis), allowing tissue repair [51]. Regarding omega-3 supplementation, the effects varied depending on the study. In the research conducted by Yang et al. [46], the authors observed an increase in TNF-α and IL-6. However, this study had a duration of 7 days. However, a significant decrease was documented in other studies [37,41], where the supplement was consumed for longer periods of time. However, there are other studies selected in this review according to inclusion criteria documenting a reduction in TNF-α and IL-6 values [45,52,55].

CRP production is stimulated by IL-6, IL-2, and TNF-α, thus being considered an indicator of acute systemic inflammation. CRP values increase in response to training [37]. The inflammatory response favors subsequent muscle repair and regeneration. In this context, two studies [37,41] indicated a significant decrease in CRP, and one [45] did not present significant variations, compared to the CG. In this line, in these two studies [37,41], the CG displayed higher values of CRP; meanwhile, in the study by Ramos-Campo et al., [45] no significant changes were noticed. Therefore, more evidence is needed to establish a clear relationship between the effect of omega-3 supplementation and CRP circulating levels.

Di Lorenzo et al. [52], in a 28-day intervention with 2 g/d of omega-3, recorded a decrease in CK and IL-6 values, with no effect on DOMS. However, in the study by Gray et al. [56], no effect on IL-6 was observed. IL-2, an interleukin that is also elevated in inflammatory response, in the research of Yang et al. [46] presented a significant decrease after 6 h. Boit et al. [57], in their research with 37 young athletes, found that krill oil supplementation, at a dose of 2 g/d for 6 weeks, reduced plasma IL-2, following strenuous exercise. IL-1β studied by Ramos-Campo et al. [45], significantly decreased at 24 h, which could be associated with a decrease in inflammation favoring recovery. Despite this, in Barquilha et al. [37], there were no significant changes. Altogether, these results indicate that more scientific research is needed to assess the role of omega-3 supplementation in the modulation of inflammation messengers.

### 4.3. Muscle Damage

In the recovery process, an acute period is distinguished, which is established in the first 96 h post-exercise [58]. In EIMD situations, omega-3 can integrate into the cell membrane and perform a protective effect [50] by decreasing muscle soreness. Omega-3 improves the integrity of the muscle membrane, preventing the leakage of intramyocellular proteins such as CK [59].

On the other hand, it should be considered that eccentric exercise leads to an increase in blood concentrations of CK, LDH, and myoglobin [46], reaching a maximum level at 1–2 days after exercise [47]. This increase is due to lipid peroxidation, which causes an increase in membrane permeability, which favors leakage to the plasma of these proteins at the end of exercise, observed at 24, 48, and 72 h [34].

Regarding CK, published data are inconsistent. One study [40] indicated increases in circulating CK values in both groups (supplemented and CG). Nevertheless, others [35,37] showed a significant decrease in CK levels from the start of the exercise in the supplemented group compared to CG; meanwhile, no differences were documented in other studies [16,17,34]. Therefore, only three studies [34,35,37] showed that the CG had significantly higher values of CK, which could be associated with greater inflammation and EIMD. The disparity in values can be explained by the type of intervention performed. Studies with high values and no significant differences with CG performed physical exercises that involve the legs and a duration of one day [40]. The variability in the results makes it difficult to establish an accurate recommendation regarding the dose and duration of supplementation.

LDH was analyzed by two studies [35,37], showing that the CG presented significantly higher values of circulating LDH than the supplemented group. Another investigation [46] showed no changes in LDH circulating levels. However, in the same publication [46], CK values decreased in the supplemented group. These discrepancies in both muscle markers (LDH and CK) could be explained by the different half-lives that these proteins display in circulation, being shorter for CK (12h) than for LDH (5–6 days).

DOMS analysis presented different results. This could be due in part to the body segments analyzed. Some studies analyzed pain in the upper part of the body [16,17,34] or involved the whole body with a preponderance of the legs [35,40]. Nevertheless, the CG presented higher values with respect to the supplemented group in all studies with the exception of one study [40]. Differences were also present throughout the measurements. Decreases in DOMS values were recorded in the supplemented group in the first 24 h [40] and 48 h after the exercise actions [35]. However, in the study by Tsuchiya et al. [34], DOMS values remained significantly elevated during the first four days in the supplemented group. Low DOMS values could be related to better performance suggesting that a supplemental intake of omega-3 could be beneficial [45]. In this line, the study by Lembke et al. [16] supplemented 65 men and women for one week with 2.7 g/d for 30 days, observing a decrease in DOMS.

Similarly, in the study by Tartibian et al. [24], supplementation with a dose of 1.8 g/d of omega-3 for 30 days decreased soreness. In the same line, Black et al. [11] supplemented for 5 weeks 20 rugby players with 1102 mg EPA and 1102 mg DHA per day, also reporting positive results on decreased muscle soreness. However, the study by Gray et al. [56] indicated no changes. The systematic review conducted by Chalchat et al. [60] analyzed the relationship between DOMS and MVC. This report established that the DOMS peak could occur late. However, discrepancies in the results are related to the fact that DOMS could depend on the level of inflammation, which may vary according to the level of loss of MVC. Therefore, the time for pain appearance will vary. In another systematic review [61], a decrease in DOMS with omega-3 supplementation was reported, but with a smaller effect size, and therefore it was not considered clinically relevant.

As myoglobin did not show differences between groups or over time, it could not be associated with the potential benefits of omega-3 consumption. However, myoglobin displays shorter half-lives in circulation (2–3 h).

### 4.4. Oxidant Response

Post-exercise condition displays an increased production of ROS, indicating that the antioxidant capacity influences the recovery process. However, ROS production depends on duration and intensity, with greater oxidative stress in longer and high-intensity exercises [55]. Antioxidant supplements are necessary when the endogenous antioxidant capacity is overwhelmed by excessive production of free radicals. In this context, omega-3 supplements decrease circulating oxidative stress markers [62], improving mitochondrial function (free radical source) and reducing inflammation by stimulating pro-inflammatory cytokine production. Altogether, this results in less muscle damage [37]. EPA and DHA can alter lipid membranes, produce disruption of lipid rafts, and inhibit NF-κβ activity, reducing the expression of pro-inflammatory genes [63]. Therefore, EPA and DHA act as antioxidants due to their influence on neutrophils, macrophages, and mediators of acute inflammation, such as resolvins and protectins [53]. EPA and DHA also inhibit the cyclooxygenase and lipoxygenase pathways from arachidonic acid (omega-6) metabolism, which is related to the inflammatory process [64].

EPA and DHA are associated with low inflammation and muscle damage markers, decreasing muscle soreness [45]. Some indicators used include TAC (total antioxidant capacity), MDA (malonyl dialdehyde) as a marker of lipid peroxidation, and oxidation markers in other macromolecules, such as proteins and DNA [65]. In this line, MDA correlates with ROS-mediated membrane oxidation [65] and TAC is the antioxidant capacity of the system, which results from the activation of antioxidant enzymes, such as superoxide dismutase (SOD). All these parameters are paramount in the EIMD recovery process [46]. The increase in MDA indicates the action of free radicals in lipid peroxidation. MDA levels decrease with omega-3 supplementation, suggesting the antioxidant capacity of the supplement, verified by increased SOD activity and TAC values [46]. In the same line, the study by Berge et al. [66] found that krill oil supplementation in 17 young athletes for 28 days significantly improved plasma antioxidant capacity, inflammation, and circulating lipid profile. In addition, Marques et al. [67] observed that 30-day supplementation with omega-3 in wheelchair athletes decreased circulating LDH and IL-6 levels, and prevented the loss of membrane integrity and mitochondrial membrane potential in neutrophils. Furthermore, Atashak et al. [68] reported a significant decrease in CK and CRP after one week of supplementation. The CG in this study displayed increased levels of MDA, that remained elevated 24 h after. Altogether, these studies indicate the antioxidant potential of omega-3 supplements, primarily acting in lipid environments, which helps maintain the integrity of cell membranes.

In this line, DHA accumulates in the phospholipid membrane and in mitochondrial cardiolipin, a membrane-bound lipid involved in the regulation of apoptosis and protection of mitochondria against oxidative stress [69]. Thus, DHA can stimulate endogenous antioxidant systems by activating signaling pathways that prevent DNA damage, as indicated by decreased levels of 8-hydroxy-2′-deoxyguanosine [69]. SOD is an antioxidant enzyme, located in the cytosol and mitochondria [21], which acts as the first line of defense against oxidative stress induced by exercise [46]. Yang et al. [46] showed high SOD levels after EIMD. However, SOD levels can be conditioned by physiological situations, such as the menstrual phase in active women. In this line, the study by McKinley et al. [70] did not observe differences in SOD concentration during the recovery period of women during the mid-luteal phase and found higher SOD concentrations in the mid-follicular phase. Luostarinen et al. [71] and Fisher et al. [72] showed that the decrease in superoxide production does not involve the cyclooxygenase pathway, suggesting a role for SOD. On the other hand, Gray et al. [56] analyzed the effect of supplementation for 6 weeks on 20 men, showing an improvement in oxidative stress indicators, although they reported no difference in DOMS and circulating CK levels. Similarly, the study by Lenn et al. [73] did not show changes in oxidative markers or CK, IL-6, or TNF-α after fish oil supplementation. This suggests that, in addition to SOD, other intracellular antioxidants are modulated by omega-3 supplements.

GSH, like SOD, is a key antioxidant that reduces oxidative stress produced by exercise [46]. Intracellular concentration of GSH is an indicator of redox status. In this context, the ratio between the reduced and the oxidized form (GSH/GSSG) is a very useful indicator of intracellular redox potential [74]. In this context and according to Barquilha et al. [37], GSH and GSH/GSSG ratios were higher in the omega-3-supplemented group.

### 4.5. Sports Performance

Physical exercise involves the breakdown of muscle myofibrils, which cause DOMS, inflammation, and muscle swelling [75]. This process results in a limited range of motion and decreased muscle strength [76]. In one study [34], there was an increase in elbow range of motion (ROM) that persisted up to 3 days after the execution of an eccentric exercise, in the omega-3 supplemented group. Similarly, another study [17] showed a significant increase in elbow ROM up to 5 days after eccentric exercise. In this study, supplementation with 600 mg/day of EPA and 260 mg/day of DHA was conducted for 8 weeks. Nevertheless, less time (4 weeks) seems to be enough to reach a similar goal [34]. Altogether, EPA and DHA supplementation benefits joint flexibility, protecting against eccentric exercises [77].

Regarding other biomechanics abilities related to performance, volunteers supplemented with omega-3 were allowed to recover baseline vertical jump performance after 24 h compared to the not-supplemented group [40]. Otherwise, higher supplementation doses had a positive impact on vertical jump recovery, an assessment of muscle power, 24 h post-exercise and longer.

In the same line, positive effects were also observed in the study by Tsuchiya et al. [17]. This study verified that omega-3 supplementation had a positive effect on maximal voluntary isometric contraction torque after eccentric exercise. However, the effects of supplementation are controversial. In some studies, no effect of omega-3 supplementation was observed on DOMS reduction, with 2 g/day of DHA for 28 days [52] or with 1.8 g/day of omega-3 for 30 days [73]. These observations suggest that longer treatments seem to be more effective in delaying DOMS [17]. In this context, DOMS, as well as inflammation, can have a negative effect on athletes in the following days post-exercise, particularly if it is necessary to execute intense activity in a short period of time. Altogether, these studies [17,34,35] indicate that omega-3 supplementation can prevent and alleviate muscle damage after eccentric exercise.

## 5. Limitations and Strengths

While some studies were found, many of them did not comply with the inclusion criteria, and therefore, had to be excluded from the systematic review. Additionally, it is important to acknowledge that the studies included in the analysis had relatively small sample sizes. Consequently, it is necessary to consider the variations in participant populations, including their different levels of training and the utilization of diverse research protocols. These factors contribute to increased heterogeneity among studies, emphasizing the need for caution when interpreting the findings. The studies included in the systematic review utilized varying doses and durations of omega-3 supplementation. This variability among studies makes it difficult to reach clear conclusions.

Nevertheless, to ensure a rigorous approach, the systematic review adhered to the PERSiST rules [28], the PICOS [29] model, and conducted a comprehensive search across three databases, Medline, Scopus, and WOS, to encompass a wide range of relevant literature. Furthermore, the methodological quality of the selected studies was assessed using the McMaster [30] assessment tool and PEDro [31], together with Cochrane’s risk of bias [32] assessment tool to ensure that all selected records met minimum quality.

Studies in this systematic review did not exclusively focus on elite athletes. Instead, they encompassed both athletes and normal healthy individuals, providing sufficient evidence to support their hypothesis. The main findings suggest that omega-3 fatty acids could potentially be accepted as a beneficial supplementation for elite athletes on the topic of muscle recovery. Nevertheless, more research could be done on omega-3 supplementation and improvement of athletic performance.

## 6. Practical Applications

Interest in finding nutrients and supplements that can enhance athletic performance and aid in recovery has witnessed significant growth in recent years. Athletes frequently employ various supplements to potentially augment their metabolic capacity, delay the onset of fatigue, promote muscle hypertrophy, and expedite recovery. Ergogenic aids have emerged as potential facilitators of these desired outcomes and omega-3, with its notable properties, has recently gained recognition as an ergogenic supplement that may contribute to some of these processes [78]. The use of nutritional aids that allow athletes to improve their health and increase their sports performance must be consistent with the principles of rational nutrition and, when necessary, do not cover nutritional requirements with diet alone [79].

Supplementation with omega-3 fatty acids has undergone extensive research, although most of the studies included a small sample size. However, all the studies analyzed in this systematic review consistently indicate that omega-3 supplementation, attributed to its anti-inflammatory properties, confers benefits in terms of muscle recovery following EIMD, as evidenced by decreased inflammatory markers, preventing oxidation of muscle satellite cells and muscle damage [80]. While these findings demonstrate promise for omega-3 supplementation and muscle recovery, the outlook for its impact on improving athletic performance is less encouraging. Limited studies were available, and the consensus among them suggests little to no evidence supporting the notion that omega-3 supplementation provides any discernible benefits in enhancing athletic performance.

Further investigation is necessary to establish a conclusive understanding of the connection between omega-3 fatty acids supplementation and muscle recovery, especially between omega-3 and athletic performance. It would be valuable to conduct multiple studies incorporating different dosages of omega-3 to different groups, participants with a similar age range, and similar exercise levels. These studies should also examine key parameters to enhance the administration methods of omega-3 based on factors such as age, gender, level of physical activity, and heart health. By doing so, an optimal and more precise range of omega-3 consumption can be determined to derive its benefits.

## 7. Conclusions

Based on the results, this systematic review showed that overall, more evidence is needed to give clear recommendations on the dose and duration of omega-3 treatment. Consumption of (at least) 2400 mg/day of omega-3 fatty acids (EPA/DHA) for (at least) 4.5 weeks appears to be effective. However, future studies have to indicate certain aspects to increase the reliability of the research, such as baseline omega-3 status matching between control and intervention groups, reporting of habitual dietary intake of omega-3 food sources, and inclusion of at least one outcome measure that validates omega-3 status change in response to supplementation. Omega-3 supplementation could benefit by reducing the inflammatory response after exercise, evidenced by IL-6 decrease. In addition, the effect that omega-3 has on EIMD is evidenced by the reduction of circulating muscle biomarkers, such as CK and LDH. Omega-3 may help decrease oxidative stress caused by exercise. Overall, the evidence shows that EPA/DHA have the potential to decrease the production of inflammatory cytokines and markers of muscle damage. However, the only study that employed ALA reported no benefit for healthy physically active subjects.

## Figures and Tables

**Table 1:** Results of the methodological quality assessment of included studies—McMaster Critical Review Form for Quantitative Studies [30].

Study	Items																Total	%	Quality Score
	1	2	3	4	5	6	7	8	9	10	11	12	13	14	15	16			
**Ávila-Gandía et al., 2020** [36]	1	1	1	1	1	0	1	1	1	1	1	1	0	1	1	0	13	81.3	VG
**Barquilha et al., 2023** [37]	1	1	1	1	0	1	1	1	1	1	1	1	1	1	1	1	15	93.8	E
**Brook et al., 2021** [38]	1	1	1	1	1	0	1	1	1	1	1	1	1	1	1	1	15	93.8	E
**Heileson et al., 2023** [39]	1	1	1	0	1	0	1	1	1	1	1	1	0	1	1	1	13	81.3	VG
**Jakeman et al., 2017** [40]	1	1	1	1	1	0	1	1	1	1	1	1	1	0	1	1	14	87.5	VG
**Lee et al., 2022** [41]	1	1	1	0	1	0	1	1	1	1	1	1	1	1	0	1	13	81.3	VG
**Lembke et al., 2014** [16]	1	1	1	1	0	1	1	1	1	1	1	1	1	1	1	1	15	93.8	E
**Mullins et al., 2022** [42]	1	1	1	1	0	1	1	1	1	1	1	1	0	1	0	1	13	81.3	VG
**Nieman et al., 2015** [43]	1	1	1	0	1	1	1	1	1	1	1	1	1	1	0	0	13	81.3	VG
**Tomczk et al., 2024** [33]	1	1	1	0	1	1	1	1	1	1	1	1	0	1	0	1	13	81.3	VG
**Tsuchiya et al., 2021** [34]	1	1	1	1	1	0	1	1	1	1	0	1	1	1	0	1	13	81.3	VG
**Tsuchiya et al., 2016** [17]	1	1	1	1	1	1	1	1	1	1	1	1	1	1	0	1	15	93.7	E
**VanDusseldrorp et al., 2020** [37]	1	1	1	1	1	1	1	1	1	1	1	1	1	0	1	1	15	93.7	E

Abbreviations: 0 = not fulfilled criterion; 1 = fulfilled criterion; E = excellent; VG = very good; Item 1: study purpose; item 2: literature review; item 3: study design; item 4: blinding; item 5: sample description; item 6: sample size; item 7: ethics and consent; item 8: validity of outcomes; item 9: reliability of outcomes; item 10: intervention description; item 11: statistical significance; item 12: statistical analysis; item 13: clinical importance; item 14: conclusions; item 15: clinical implications; item 16: study limitations.

**Table 2:** Methodological quality according to the PEDro Scale [31].

Study	Items											Total	%	Quality Score
	1	2	3	4	5	6	7	8	9	10	11			
**Ávila-Gandía et al., 2020** [36]	1	1	1	1	1	1	0	1	1	1	1	10	90.9	E
**Barquilha et al., 2023** [37]	1	1	1	1	1	1	0	1	1	1	1	10	90.9	E
**Brook et al., 2021** [38]	1	1	1	1	1	1	0	1	1	1	1	10	90.9	E
**Heileson et al., 2023** [39]	1	1	1	1	0	1	0	1	1	1	1	9	81.8	E
**Jakeman et al., 2017** [40]	1	1	1	1	1	1	0	1	1	1	1	10	90.9	E
**Lee et al., 2022** [41]	1	1	1	1	0	0	0	1	1	1	1	8	72.7	G
**Lembke et al., 2014** [16]	1	1	1	1	1	0	0	1	1	1	1	9	81.8	E
**Mullins et al., 2022** [42]	1	1	1	1	1	1	0	1	1	1	1	10	90.9	E
**Nieman et al., 2015** [43]	0	1	1	1	0	1	0	1	1	1	1	8	72.7	G
**Tomczk et al., 2024** [33]	1	1	1	1	0	0	0	1	1	1	1	8	72.7	G
**Tsuchiya et al., 2021** [34]	1	1	1	1	1	1	0	1	1	1	1	10	90.9	E
**Tsuchiya et al., 2016** [17]	0	1	1	1	1	1	0	1	1	1	1	9	81.8	E
**VanDusseldrorp et al., 2020** [37]	1	1	1	1	1	1	0	1	1	1	1	10	90.9	E

Abbreviations: E = Excellent; G = Good; 1 = Criterion met; 0 = Criterion not met, Item 1 = whether the selection criteria were specified; 2 = subjects were randomly assigned to groups; 3 = allocation was hidden; 4 = the groups were similar at baseline in relation to the most important prognostic indicators; 5 = subjects were blinded; 6 = therapists administering therapy were blinded; 7 = assessors were blinded; 8 = at least one of the key outcomes its measures were obtained from more than 85% of the assigned subjects; 9 = results were presented for all subjects who received treatment or were assigned to the control group, or when this could not be, data for at least one key outcome were analyzed by ‘intention to treat; 10 = results of statistical comparisons between groups were reported for at least one key outcome; 11 = point and variability measures of at least one key outcome were provided.

**Table 3:** Cochrane Risk of Bias Assessment [32].

	random sequence generation (selection bias)	allocation concealment (selection bias)	blinding (performance bias and detection bias) participant	blinding (performance bias and detection bias) personnel	blinding (performance bias and detection bias) outcome assessor	incomplete outcome (attrition bias)	selective reporting (reporting bias)	other bias
Ávila-Gandía et al., 2020 [36]	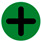	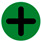	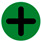	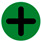	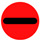	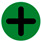	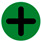	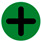
Barquilha et al., 2023 [37]	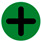	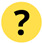	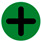	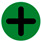	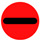	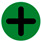	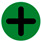	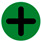
Brook et al., 2021 [38]	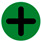	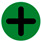	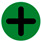	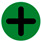	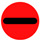	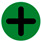	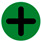	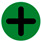
Heileson et al., 2023 [39]	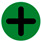	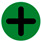	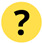	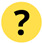	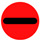	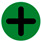	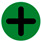	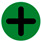
Jakeman et al., 2017 [40]	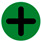	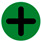	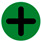	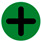	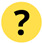	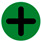	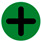	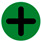
Lee et al., 2022 [41]	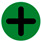	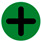	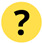	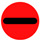	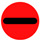	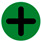	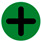	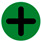
Lembke et al., 2014 [16]	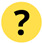	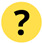	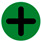	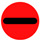	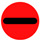	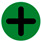	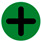	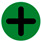
Mullins et al., 2022 [42]	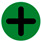	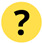	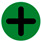	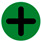	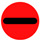	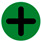	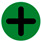	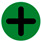
Nieman et al., 2015 [43]	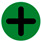	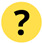	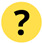	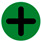	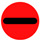	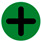	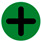	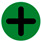
Tomczk et al., 2024 [33]	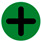	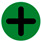	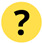	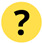	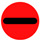	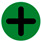	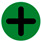	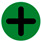
Tsuchiya et al., 2021 [34]	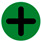	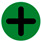	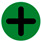	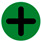	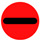	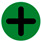	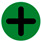	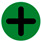
Tsuchiya et al., 2016 [17]	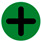	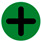	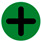	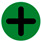	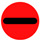	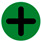	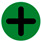	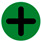
VanDusseldrorp et al., 2020 [37]	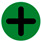	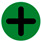	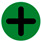	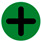	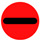	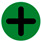	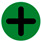	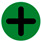

Risk of bias summary: review authors’ judgments about each risk of bias item for each included study. (+) = low risk of bias; (?) = unclear risk of bias; (−) = high risk of bias.

**Table 4:** Characteristics of participants and supplementation protocols of the selected studies.

Characteristics	Types	Reference
**Level of participants**	Amateur competitive	[36,42,43]
	Amateur	[33]
	Recreationally	[17,33]
	Recreationally active	[38,39]
	Physically active	[16,35,37,40,41]
**Administration Type**	Capsule	[16,17,33,34,36,38,39,40]
	Soft gel	[35,42]
	Water with seed oil	[43]
	Unspecified	[33,38]
**Total dose**	High * (750 mg EPA + 50 mg DHA) Low * (150 mg EPA + 100 mg DHA)	[40]
	2 g (1400 mg: 800 mg EPA + 600 mg DHA) 4 g (2800 mg: 1600 mg EPA + 1200 mg DHA) 6 g (4200 mg: 2400 mg EPA + 1800 mg DHA)	[35]
	780 mg EPA + 606 mg DHA	[37]
	1220 mg/d (975 mg DHA + 120 mg EPA)	[36]
	2.1 g/d EPA + 0.78 g/d DHA	[41]
	2.275 g/d EPA + 1.575 g/d DHA	[39]
	2234 mg/d EPA + 930 mg/d DHA	[33]
	2.4 g/d (600 mg EPA + 260 mg DHA)	[34]
	2.7 g/day	[16]
	3.5 g/d (1 g: 407 mg/g DHA +170 mg/g EPA)	[43]
	2400 mg (1360 mg EPA + 1040 mg DHA)	[17]
	3680 mg/d (1860 mg EPA +1540 mg DHA)	[38]
	31 g ALA for the average	[32]
**Dose schedule**	once a day: post-lunch morning	[34]
	30 min after meals with water	[17]
	30 min before exercise	[43]
	once a day: post-exercise	[40]
	3 times/days (morning, lunch, dinner)	[41]
	Unspecified	[16,33,35,36,37,38,39,42]
**Amount of supplement/d**	2/4/6 capsules	[35]
	3 capsules	[36,37,41]
	6 capsules	[16,42]
	8 capsules	[17,34]
	1 g (capsule)/10 kg/BM	[40]
	7 capsules	[39]
	0.43 g ALA/kg BM	[43]
	Unspecified	[38]
**Duration**	twice separated by two weeks	[43]
	1 day	[40]
	30 days	[36]
	4 weeks	[16]
	4.5 weeks	[34]
	6 weeks	[37,38]
	7.5 weeks	[35]
	8 week + 5 days	[17]
	10 weeks	[39]
	12 weeks	[33,41]
	26 weeks	[42]
**Exercise intervention**	Endurance + functional/resistance	[33,35]
	Cycling test to exhaustion	[36]
	Maximum eccentric extensions of the forearm or elbow	[16,17,34]
	Plyometric jumps	[40]
	Resistance exercise training	[37,38,39,41]
	Running at constant speed until exhaustion	[43]
	Unspecified	[42]

*Abbreviations:* ALA = alpha-linolenic acid; BM = body mass; DHA = docosahexaenoic acid; EPA = eicosapentaenoic acid; * 1 g/10 kg BM; mg = milligrams; g = grams; d = day; kg = kilograms.

**Table 5:** Studies included in the systematic review of the effect of Omega-3 on inflammatory markers, oxidant response, muscle damage, and physical performance in healthy and physically active adults.

First Author, Year of Publication, and Country	Study Design	Participants	Intervention	Outcomes	Results	
Ávila-Gandía et al. [36], 2020, Spain	Randomized, double-blind, placebo-controlled, parallel-group trial	*n* = 50 ♂ Amateur cyclists competing at regional level Gn-3 *n* = 18 Age (mean ± SD) 35.5 ± 7.3 years Weight (mean ± SD) t 72.4 ± 4.4 kg BMI (mean ± SD) 23.83 ± 1.43 Relative VO_2_ max (mean ± SD) 48.5 ± 6.8 mL/min/kg CG *n* = 20 Age (mean ± SD) 36.0 ± 9.6 years Weight (mean ± SD) 71.1 ± 3.4 kg BMI (mean ± SD) 23.42 ± 1.31 Relative VO_2_ max (mean ± SD) 49.3 ± 6.1mL/min/kg Study withdrawals: 12	Gn-3 3 soft-gels Per unit: 325 mg DHA + 40 mg EPA (Brudy plus, Brudytechnology, Barcelona, Spain) CG Sunflower oil Supplementation time: 30 days	Muscle damage Blood Lactate Physical performance Absolute VO_2_ HR MPO Relative VO_2_ RP time VO_2_ VT2	Gn-3 vs. CG ↔ Blood Lactate ↓*Absolute VO_2_ (6’) ↓* HR ↑* MPO ↓* Relative VO_2_ (6’) ↑* RP ↑* Time ↔ VO_2_ ↑* VT2	Gn-3 Changes from baseline ↔ Blood Lactate ↓* Absolute VO_2_ (6’) ↓* HR ↑* MPO ↓* Relative VO_2_ (6’) ↑* RP ↑* Time ↑* VO_2_ CG Changes from baseline ↔ Blood Lactate ↔ Absolute VO_2_ (6’) ↔ HR ↔ MPO ↔ Relative VO_2_ (6’) ↔ RP ↔ time ↔ VO_2_
Barquilha et al. [37], 2023, Brazil	Randomized, double-blind, placebo-controlled, parallel-group trial	*n* = 21 ♂ Gn-3 *n* = 8 CG *n* = 8 Physically active Age: 20–30 years Study withdrawals: Gn-3 *n* = 3 CG *n* = 2	Gn-3 3 capsules Per unit: 260 mg EPA + 202 mg DHA 3 times daily (Capsule Naturalis Nutricao & Farma LTDA, Sao Paulo, Brazil) Supplementation time: 6 weeks	Hematology Heme Iron Iron Hormones T/C Inflammatory biomarkers CRP IL-6 Muscle damage CK LDH Oxidative stress GSH GSSG GSH/GSSG TEAC	Gn-3 vs. CG ↔ Heme Iron ↔ Iron ↓ CRP ↓ IL-6 ↓ CK ↓ LDH ↑* GSH ↓* GSSG ↑* GSH/GSSG ↔TEAC	*Gn-*3 Changes from baseline ↔T/C ↓* CRP ↓* IL-6 ↓ CK ↓ LDH
Brook et al. [38], 2021, United Kingdon	Randomized, double-blind, placebo-controlled, parallel-group trial	*n* = 16 ♀ Recreationally active Gn-3 *n* = 8 ♀ Age (mean ± SD) 64.4 ± 0.8 years Height (mean ± SD) 162 ± 0.02 cm Weight (mean ± SD) 70.5 ± 2.5 kg BMI (mean ± SD) 26.6 ± 0.7 kg/m^2^ % Fat (mean ± SD) 40.8 ± 1.1% Lean Mass (mean ± SD) 39.4 ± 1.1 kg CG *n* = 8 ♀ Age (mean ± SD) 66.5 ± 1.4 years Height (mean ± SD) 158 ± 0.02 cm Weight (mean ± SD) 64.3 ± 1.9 kg BMI (mean ± SD) 2.8 ± 0.9 kg/m^2^ % Fat (mean ± SD) 39.1 ± 1.6% Lean Mass (mean ± SD) 37.1 ± 1.6 kg Study withdrawals: 0	Gn-3 Per unit: 1860 mg EPA +1540 mg DHA (Minami Epacor) CG Cornoil Supplementation time: 6 weeks	Anthropometry BM Bone mass FFM LBM Muscle function ASR Calpain MAFbx MPS Myonuclei SC Ubiquitin VL Physical performance 1-RM MVC	Gn-3 vs. CG ↔ BM ↔ Bone mass ↔ FFM ↔ LBM ↔ ASR _untrained leg (0–6 weeks)_ ↔ Calpain ↔ MAFbx ↔ MPS _untrained leg_ ↔ Myonuclei _type I-II fibre_ ↔ SC _type I fibre_ ↔ Ubiquitin ↑ 1-RM _trained leg_ ↔ MCV _trained leg_ ↔ MCV _untrained leg_	Gn-3 Changes from baseline ↔ BM ↔ Bone mass ↔ FFM ↔ LBM ↑* ASR _untrained leg (0–2 weeks)_ ↑ ASR _untrained leg (4–6 weeks)_ ↔ Calpain ↔ MAFbx ↔ MPS _untrained leg (0–2 weeks)_ ↔ MPS _untrained leg (0–4 weeks)_ ↑* Myonuclei _type I-II fibre_ ↔ SC _type I fibre_ ↔ Ubiquitin ↑ 1-RM _trained leg_ ↔ MCV _trained leg_ ↔ MCV _untrained leg_ CG Changes from baseline ↔ BM ↔ Bone mass ↔ FFM ↔ LBM ↑* ASR _untrained leg (0–2 weeks)_ ↔ ASR _untrained leg (4–6 weeks)_ ↔ Calpain ↔ MAFbx ↑* MPS _untrained leg (0–2 weeks)_ ↔ MPS _untrained leg (2–4 weeks)_ ↑* Myonuclei _type I-II fibre_ ↔ SC _type I fibre_ ↔ Ubiquitin ↑ 1-RM _trained leg_ ↔ MCV _trained leg_ ↔ MCV _untrained leg_
Heileson et al. [39], 2023, United States	Randomized, single-blind, placebo-controlled, parallel-group trial	*n* = 28 (*n* = 12 ♂ and *n* = 16 ♀) Recreationally Trained Gn-3 *n* = 10 *n* = 5 ♂ and *n* = 5 ♀ Age (mean ± SD) 28.0 ± 7.4 years Height (mean ± SD) 169.7 ± 9.6 cm Weight (mean ± SD) 75.1 ± 16.0 kg BMI (mean ± SD) 25.8 ± 3.5 kg/m^2^ % Fat (mean ± SD) 23.9 ± 6.9% CG *n* = 11 5 ♂ and 6 ♀ Age (mean ± SD) 30.5 ± 5.7 years Height (mean ± SD) 171.8 ± 8.9 cm Weight (mean ± SD) 79.0 ± 16.0 kg BMI (mean ± SD) 26.6 ± 4.3 kg/m^2^ % Fat (mean ± SD) 24.9 ± 8.0% Study withdrawals: Gn-3 *n* = 4 CG *n* = 3	Gn-3 7 capsules 2.275 g/d EPA + 1.575 g/d DHA (Nordic Naturals, ProOmega, Watsonville, CA, USA) CG 5 capsules 4.5 g/d (NOW, Bloomingdale, IL, USA) Supplementation time: 10 weeks	Anthropometry LBM FM BF Biochemistry DBS Physical performance absolute 1RM_BP_ absolute 1RM_SQT_ ∆ relative 1RM_BP_ ∆ relative 1RM_SQT_	Gn-3 vs. CG ↔ LBM ↔ FM ↔ BF ↑* DBS ↑* absolute1RM_BP_ ↔ absolute 1RM_SQT_ ↑* ∆ relative 1RM_BP_ ↑* ∆ relative 1RM_SQT_	Gn-3 Changes from baseline ↑ LBM ↓ FM ↓ BF ↑* DBS ↑ absolute 1RM_BP_ ↑ absolute 1RM_SQT_ ↑* ∆ relative 1RM_BP_ ↑* ∆ relative 1RM_SQT_ CG Changes from baseline ↑ LBM ↓ FM ↔ BF ↔ DBS ↑ absolute 1RM_BP_ ↑ absolute 1RM_SQT_ ↑* ∆ relative 1RM_BP_ ↑* ∆ relative 1RM_SQT_
Jakeman et al. [40], 2017, United Kingdom	Randomized, double-blind, placebo-controlled, parallel-group trial	*n* = 27 ♂ Physically active > 3 h/week of vigorous athletic training + HIIT High Gn-3 *n* = 9 Age (mean ± SD) 25.5 ± 5.2 years Height (mean ± SD) 1.74 ± 0.06 m Weight (mean ± SD) 76.5 ± 12.6 kg Low Gn-3 *n =* 9 Age (mean ± SD) 25.6 ± 4.8 years Height (mean ± SD) 1.82 ± 0.09 m Weight (mean ± SD 80.2 ± 12.0 kg CG *n* = 9 Age (mean ± SD) 26.2 ± 4.2 years Height (mean ± SD) 1.78 ± 0.01 m Weight (mean ± SD 82.9 ± 12.1 kg Study withdrawals: 0	Gn-3 1 g/capsule Dose: 1 g/10 kg BM High Gn-3 (EPA 750 mg + DHA 50 mg)/capsule Low Gn-3 (EPA 150 mg + DHA 100 mg)/ capsule CG Oil (flavour masker and gelatine) Supplementation time: 1 day	Inflammatory biomarkers IL-6 Muscle damage CK Perception markers VAS Physical performace CJ Knee extensor strength SJ	High Gn-3, Low Gn-3 vs. CG ↔ IL-6 ↔ CK ↔ VAS ↔ CJ ↔ Knee extensor strength ↑* SJ	High Gn-3, Low Gn-3 Changes from baseline ↔ IL-6 ↑* CK (24 h) ↑* VAS (24 h) ↓ (at 96 h) ↓ CJ (at 1 h) ↓* Knee extensor strength to 60° s^−1^ and 180° s^−1^(1 h–96 h) ↓* SJ (at 1 h)
Lee et al. [41], 2022, United States	Randomized, placebo-controlled trial	*n* = 28 (*n* = 10 ♂ and *n* = 18 ♀) Physically active RET-G n-3 *n* = 10 Age (mean ± SD) 67.1 ± 4.4 years Height (mean ± SD) 171.6 ± 9.3 cm Weight (mean ± SD) 70.8 ± 13.5 kg BMI (mean ± SD) 24.0 ± 3.2 kg/m^2^ RET *n* = 10 Age (mean ± SD) 66.6 ± 7.3 years Height (mean ± SD) 167.9 ± 5.7 cm Weight (mean ± SD) 66.5 ± 11.5 kg BMI (mean ± SD) 23.5 ± 3.6 kg/m^2^ CG *n* = 8 Age (mean ± SD) 66.5 ± 5.0 years Height (mean ± SD) 167.2 ± 10.24 cm Weight (mean ± SD) 68.9 ± 15.8 kg BMI (mean ± SD) 24.3 ± 3.4 kg/m^2^ Study withdrawals: 0	RET- Gn-3: 3 capsules/day Per unit: 700 mg EPA + 240 mg DHA RET 3 capsules/day Safflower oil CG 3 capsules/day Safflower oil Supplementation time: 12 weeks	Inflammatory biomarkers IL-6 CRP TNF-α Metabolism TMR FAT oxidation CHO oxidation Physical Performance 1RM lat pull-dow 1RM leg-press 1RM seated row 1RM calf rise 1RM biceps curl VO_2_ VCO_2_ RER	RET-Gn-3 vs. RET vs. CG ↓* IL-6 (RET-Gn-3 vs. CG) ↓* CRP ↓* TNF-α (RET-Gn-3 vs. CG) ↔ TMR ↑* 1RM lat pull-dow (RET-Gn-3, RET) ↑* 1RM leg-press (RET-Gn-3, RET) ↑* 1RM seated row (RET-Gn-3, RET) ↑* 1RM calf rise (RET-Gn-3, RET) ↑* 1RM biceps curl (RET-Gn-3, RET) ↑* VO_2_ ↑* VCO_2_ ↓* RER	RET-Gn-3 Changes from baseline ↓* IL-6 ↓* CRP ↓ TNF-α ↑* TMR ↑* FAT oxidation ↓* CHO oxidation ↑* 1RM in lateral pull ↑* 1RM leg-press ↑* 1RM seated row ↑* 1RM calf rise ↑* 1RM biceps curl ↑* VO_2_ ↑* VCO_2_ ↓* RER RET Changes from baseline ↔ IL-6 ↔ CRP↔ TNF-α ↑* TMR↑ FAT oxidation ↓ CHO oxidation ↑* 1RM in lateral pull ↑* 1RM leg-press ↑* 1RM seated row ↑* 1RM calf rise ↑* 1RM biceps curl ↑* VO_2_ ↑* VCO_2_ ↔ RER CG Changes from baseline ↔ IL-6 ↔ CRP ↑ TNF-α ↔ TMR ↔ FAT oxidation ↔ CHO oxidation ↓* 1RM in lateral pull ↓* 1RM leg-press ↔ 1RM seated row ↓* 1RM calf rise ↓* 1RM biceps curl ↔ VO_2_ ↔ VCO_2_ ↔ RER
Lembke et al. [16], 2014, United States	Randomized, Single-blind, placebo-controlled, parallel-group trial	*n* = 69 ♂ and ♀ Physically active Gn-3 *n* = 42 Age (mean ± SD) 18.6 ± 1.2 years CG *n* = 22 Age (mean ± SD) 18.9 ± 1.1 years Study withdrawals: 5	Gn-3 6 capsules 2.7 g/day (KD Pharma, Bexbach, Germany) CG 6 capsules High oleic sunflower oil Supplementation time: 30 days	Inflammatory biomarkers CRP Muscle damage Blood lactate CK Perception markers VAS POMS Physical Performance ROM Torque	Gn-3 vs. CG ↓* CRP ↓* Blood lactate ↔ CK (48 -96 h) ↓* VAS (at 72, at 96 h) ↑* POMS (72 h) ↔ ROM ↔ Torque	Gn-3 Changes from baseline ↓* CRP ↔ CK ↓ VAS ↓ POMS (at 48 h and 96 h) ↑ POMS (at 48 h, and at 96 h CG) ↓ ROM ↓ Torque (until 48 h)
Mullins et al. [42], 2022, United States	Randomized, double-blind, placebo-controlled, parallel-group trial	*n* =38 ♂ Competitive Gn-3 *n* =12 CG *n* = 17 Study withdrawals: Gn-3 *n* =7 CG *n* = 2	Gn-3 Soft gel capsules Per unit: 1 g: 407 mg/g DHA+ 170 mg/g EPA Pharmavite (West Hills, California) CG Per capsule 713 mg/g oleic acid + 130 mg/g linoleic acid (safflower oil) Pharmavite (West Hills, California) Supplementation time: 26 weeks	Biochemistry Plasma AA Plasma DHA Plasma DPA Plasma EPA Inflammatory biomarkers IL-6 TNF-α Injury Neurofilament	Gn-3 vs.CG ↔ Plasma AA ↓* Plasma DHA _(0–7 weeks)_ ↑* Plasma DHA _(8–26 weeks)_ ↓* Plasma DPA _(week 33)_ ↑* Plasma EPA_(week 8,12,17,21)_ ↔ IL-6 ↔ TNF-α ↔ Neurofilament	Gn-3 Changes from baseline ↓*Plasma AA _(week 8,12,17,21,26)_ ↑*Plasma DHA ↑*Plasma DPA _(week 8)_ ↑*Plasma EPA _(week 8,12,17,21,26)_ ↔IL-6 ↔TNF-α ↑Neurofilament CG Changes from baseline ↔Plasma AA ↔Plasma DHA ↔Plasma DPA ↔Plasma EPA ↔IL-6 ↔TNF-α ↑Neurofilament
Nieman et al. [43], 2015, United States	Randomized (1:1 allocation), placebo-controlled, crossover trial	*n* = 24 16 ♂ and 8 ♀ Competitive runners Age (mean ± SD) 38.0 ±1.7 year Height (mean ± SD) 1.72 ±0.02 m Weight (mean ± SD) 71.8 ± 3.0 kg % Fat (mean ± SD) 19.9 ±1.6 VO_2max_ (mean ± SD) 47.9 ±1.6 Study withdrawals: 0	Gn-3 0.5 L water with chia seed oil 0.43 g ALA/BM (Dole Foods California, USA), CG 0.5 L of flavored water alone Supplementation time: two occasions separated by 2 weeks	Biochemistry Plasma glucose Leukocyte Plasma ALA Hormones Cortisol Inflammatory biomarkers IL-6 IL-8 IL-10 TNF-α Muscle damage Blood lactate Perception markers RPE Physical performance HR RER VO_2_	Gn-3 *vs.*CG ↓ Plasma glucose ↔ Leukocyte ↑* Plasma ALA ↑* Cortisol ↓ IL-6 ↓ IL-8 ↓ IL10 ↓TNF-α ↓ Blood lactate ↓ RPE ↔ HR ↔ RER ↓VO_2_	Changes from baseline ↑ Plasma glucose ↑* Leukocyte ↑* Plasma ALA ↑* Cortisol ↑* IL-6 ↑* IL-8 ↑* IL10 ↑* TNF-α ↑ Blood lactate
Tomczyk et al. [33], 2024, Poland	Randomized, placebo-controlled, parallel-group trial	*n* = 40 ♂ Endurance runners Gn-3 *n* = 14 Age (mean ± SD) 37 ± 3 years Height (mean ± SD) 181 ± 7 cm Weight (mean ± SD) 76 ± 11 kg HRmax (mean ± SD) 190 ± 9 beats/min^−1^ CG *n* =12 Age (mean ± SD) 37 ± 4 years Height (mean ± SD) 180 ± 4 cm Weight (mean ± SD) 78 ± 8 kg HRmax (mean ± SD) 186 ± 9 beats/min^−1^ Study withdrawals: 14	Gn-3 2234 mg/d EPA + 930 mg/d DHA CG 4000 mg/d MCT Supplementation time: 12 weeks	Biochemistry Red blood cell DHA Plasma DHA Red blood cell EPA Plasma EPA Plasma Trp metabolites (7) Inflammatory biomarkers IL-6 Perception markers EA HT TA	Gn-3 vs. CG IL-6 Red blood cell DHA Plasma DHA Red blood cell EPA Plasma EPA Plasma Trp metabolites ↔ EA ↔ HT ↔ TA	Gn-3 Changes from baseline ↑* Red blood cell DHA ↑* Plasma DHA ↑* Red blood cell EPA ↑* Plasma EPA ↑* Plasma Trp metabolites ↔ IL-6 ↔ EA ↔ HT ↔ TA CG Changes from baseline ↔ Red blood cell DHA ↔ Plasma DHA ↔ Red blood cell EPA ↔ Plasma EPA ↔ Plasma Trp metabolites ↔ IL-6 ↔ EA ↔ HT ↔ TA
Tsuchiya, et al. [34], 2021, Japan	Randomized, double-blind, placebo-controlled, parallel-group trial	*n* =23 ♂ Recreational Gn-3 *n* = 11 Age (mean ± SD) 20.2 ± 0.4 years Height (mean ± SD) 167.4 ± 5.4 cm Weight (mean ± SD) 65.0 ± 8.9 kg % Fat (mean ± SD) 17.2 ± 6.9% BMI (mean ± SD) 23.2 ± 2.9 kg/m^2^ CG *n* = 11 Age (mean ± SD) 19.8 ± 1.5 years Height (mean ± SD) 169.0 ± 7.8 cm Weight (mean ± SD) 65.4 ± 8.4 kg % Fat (mean ± SD) 15.7 ± 7.6% BMI (mean ± SD) 23.2 ± 3.3 kg/m^2^ Study withdrawals: 1	Gn-3: 8 Softgel capsule of 300 mg /d Total: 2.4 g/d (600 mg EPA + 260 mg DHA) Nippon Suisan Kaisha Ltd., Tokyo, Japan CG: 8 softgel capsules of 300 mg/d corn oil Supplementation time: 4.5 weeks	Anthropometry UAC Biochemistry Blood lipids AA EPA DGLA DHA Dietary Intake Kcal CHO prot FAT Omega-3 Inflammatory biomarkers IL-6 Muscle damage CK Perception markers VAS Physical Performance Echo thickness Echo intensity MVIC ROM	Gn-3 vs. CG ↔ UAC ↑* EPA ↔ Kcal ↔ CHO ↔ prot ↔ FAT ↔ Omega-3 ↔ IL-6 ↓* CK ↔ VAS ↔ Echo thickness ↔ Echo intensity ↔ MVIC ↑* ROM (IP)	Gn-3 Changes from baseline ↑*UAC (IP) ↑* EPA (after 4 w) ↔ AA ↔ DGLA ↔ DHA ↔ Kcal ↔ CHO ↔ prot ↔ FAT ↔ Omega-3 ↔ CK ↔ IL-6 ↑* VAS (1–4 d) ↔ Echo thickness ↑ Echo intensity ↓* MVIC **ROM: G n-3:** ↓* IP and 1 d, after ↑ = to pre
Tsuchiya et al. [17], 2016, Japan	Randomized, double-blind, placebo-controlled, parallel-group trial	*n* = 24 ♂ Recreational Gn-3 *n* = 12 Age (mean ± SD) 19.4 ± 0.7 years Height (mean ± SD) 174.4 ± 5.6 cm Weight (mean ± SD) 64.3 ± 7.7 kg % Fat (mean ± SD) 13.0 ± 3.5% CG *n* =12 Age (mean ± SD) 19.5 ± 0.8 years Height (mean ± SD) 174.3 ± 6.7 cm Weight (mean ± SD) 66.2 ± 8.0 kg % Fat (mean ± SD) 13.6 ± 2.8% Study withdrawals: 0	Gn-3 8 Softgel capsule Fish oil Per unit: 300 mg EPA + 130 mg DHA (Nippon Suisan Kaisha Ltd. Tokyo) CG 8 Softgel capsule Per unit: 300 mg corn oil (Nippon Suisan Kaisha Ltd. Tokyo) Supplementation time: 8 weeks prior to exercise + 5 days after exercise	Anthropometry UAC Biochemistry AA DHA Inflammatory biomarkers IL-6 TNF-α Muscle damage CK Mb Perception markers VAS brachii VAS brachialis VAS brachioradialis Physical Performance MVC torque ROM	Gn-3 vs. CG ↔ UAC ↔ AA ↔ DHA ↓* IL-6 ↔ TNF-α ↔ CK ↔ Mb ↔ VAS brachii ↓* VAS brachialis ↔VAS brachioradialis ↑* MVC ↑* ROM	Gn-3 Changes from baseline ↔ UAC ↔ AA ↔ DHA ↔ IL-6 ↔ TNF-α ↔ CK ↔ Mb ↑ VAS brachii (at day 1–3) ↑* VAS brachialis (at day 2) ↔VAS brachioradialis ↓*MVC ↓ ROM CG Changes from baseline ↔ UAC ↔ AA ↔ DHA ↑* IL-6 (at day 3) ↔ TNF-α ↔ CK ↑* Mb ↑ VAS brachii (day 1 to day 3) ↑* VAS brachialis (day 1 to day 3) ↔ VAS brachioradialis ↓* MVC ↓* ROM (at day 3)
VanDusseldrorp et al. [35], 2020, United States	Randomized, double-blind, placebo-controlled, parallel-group trial	*n* = 32 (16 ♂ and 16 ♀) Physically active: 3 to 5 d/w, minimum of 3 h/w and a maximum of 8 h/w and no more than 2 h/w of aerobic exercise 2 g Gn-3 *n* = 8 (4♂ and 4♀) Age (mean ± SD) 23.5 ± 3.3 years Height (mean ± SD) 170.9 ± 6.9 cm Weight (mean ± SD) 76.1 ± 14.2 kg % Fat (mean ± SD) 20.8 ± 4.1% 4g Gn-3 *n* = 8 (4♂ and 4♀) Age (mean ± SD) 23.3 ± 3.0 years Height (mean ± SD) 172.9 ± 4.7 cm Weight (mean ± SD) 69.7 ± 15.9 kg % Fat (mean ± SD) 19.0 ± 6.2% 6g Gn-3 *n* = 8 (4♂ and 4♀) Age (mean ± SD) 23.8 ± 2.8 years Height (mean ± SD) 173.8 ± 7.6 cm Weight (mean ± SD) 72.8 ± 13.5 kg % Fat (mean ± SD) 19.4 ± 6.1% CG *n* = 8 (4♂ and 4♀) Age (mean ± SD) 23.0 ± 3.0 years Height (mean ± SD) 173.6 ± 6.2 cm Weight (mean ± SD) 67.9 ± 10.7 kg % Fat (mean ± SD) 20.6 ± 7.2% Study withdrawals: 0	Gn-3 Capsule Per unit: 400 mg EPA + 300 mg DHA 2 g Gn-3 2 g/d (1400 mg: 800 mg EPA + 600 mg DHA) 4 g Gn-3 4 g/d (2800 mg: 1600 mg EPA + 1200 mg DHA) 6g Gn-3 6 g/d (4200 mg: 2400 mg EPA + 1800 mg DHA) (MusclePharm, Denver, USA) CG Safflower oil (Capsule Muscle Pharm) Supplementation time: 7.5 weeks	Muscle damage CK LDH Perception markers VAS Performance MVIC VJ _height_ 40 yd Sprint	2 g Gn-3, 4g Gn-, 6g Gn-3 vs. CG 24 h: ↓*6 g Gn-3 vs. 2 g Gn-3 48 h: ↓* 6 g Gn-3 vs. 4 g Gn-3 72 h: ↓* 6 g Gn-3 vs. CG LDH ↓* 6 g Gn-3 vs. CG (at to 72 h) ↓* 6 g Gn-3 vs. 2 g Gn-3 (at to 72 h) VAS 2 h: CG ↑* vs. 6 g Gn-3 24 h: ↓* 4 g Gn-3 vs. CG ↑* CG vs. 6 g Gn-3 48 h: ↑* CG vs. 6 g Gn-3 ↓* 6 g Gn-3 vs. 4 g Gn-3, 2 g Gn-3 72 h: ↑* CG vs. 4 g Gn-3 ↑* CG vs. 6 g Gn-3 ↔ MVIC ↓* VJ _height_ CG ↔ 40 yd Sprint	Changes from baseline ↑* CK in all group ↑* LDH in all group ↓* 40 yd Sprint VAS ↓* MVIC (until 70 h) ↓ VJ _height_ (until 48 h) ↑* in all group (24 h) ↑* CG, 2 g Gn-3, 4 g Gn-3 (48 h)

*Abbreviations:* ↑ = no significant increase; ↓ = no significant decrease; ↑* = significant increase; ↓* = significant decrease; ↔* = no significant change; ↔ no change; * = indicates significant values (*p* < 0.05); Gn-3 = omega-3 supplementation group; CG = control group; *n* = number of participants; AA = arachidonic acid; ALA = alpha-linolenic acid; ASR = absolute synthesis rate; BF = body fat; BMI = body mass index; BM = body mass; CHO = carbohydrates; CK = creatin kinase; CJ = countermovement jump; CRP = C-reactive protein d = day; w = week; DBS = Fatty acid dried blood spot; DGLA = dihomo-gamma-linolenic acid; DHA = docosahexaenoic acid; DPA = docosapentaenoic acid; DOMS = perceived muscle pain; EA = Energetic arousal; EPA = eicosapentaenoic acid; FFM = fat free mass; FM = fat mass; GSH = reduced glutathione, GSSG = oxidized glutathione; GSH/GSSG = reduced/oxidized glutathione ratios; h = hours; HR = heart rate; HRmax = maximum heart rate; HIIT = high-intensity intermittent training; HT = hedonic tone; IETE = incremental exercise test to exhaustion; IL1β = interleukin 1 beta; IL-2 = interleukin-2; IL-6 = interleukin 6; IL-8 = interleukin 8; IL-10 = interleukin 10; IP = immediately post-exercise; kcal = calories; LBM = lean body mass; LDH = lactate dehydrogenase; Mb = Myoglobin; MCT = medium chain triglycerides; MPS = muscle protein synthesis; MPO = mean power output; MVIC = maximal voluntary isometric contraction; MVC = maximal voluntary contraction; POMS = profile of mood states; PUFA = omega-3 polyunsaturated fatty acids; RER = Respiratory exchange ratio; ROS = reactive oxygen species; RT = relative power; RPE = perceived exertion; RET = Resistance exercise training; ROM = range of motion; 1RM = maximum repetition; SC = satellite cell; SJ = Squat jump; TA = tense arousal; T-AOC = Total antioxidant capacity; T/C: Testosterone/cortisol; TEAC = trolox equivalent antioxidant capacity; TNFα = Tumor necrosis factor-alpha; protein = protein; TMR = resting metabolic rate; Trp = tryptophan; Pre = pre exercise; Post = post exercise; UAC = upper arm circumference; VAS: visual analogue scale; VCO_2_max = volume of carbon dioxide; VJ = vertical jump; VL = vastus lateralis;VT2 = ventilatory threshold 2; VO_2_max = oxygen volume; w = week; yd = yards.

## Data Availability

Not applicable.

## Appendix A. Search Strategy

**Database**	**Keywords**	**Hits**
**PubMed**	(omega-3 OR omega-3 supplementation OR Polyunsaturated fatty acids) AND (“muscle recovery”), AND (athletic performance OR improved athletic performance) AND (exercise-induced muscle damage OR muscle soreness OR muscle damage) AND (eccentric exercise) AND (inflammation OR oxidative stress) AND (benefits). Filters: Full text, Trial, in the last 10 years	678
**Scopus**	(“omega-3” [Title/Abstract] OR “omega-3 supplementation” [Title/Abstract] OR “Polyunsaturated fatty acids” [Title/Abstract]) AND (“muscle recovery” [Title/Abstract]), AND (“athletic performance” [Title/Abstract] OR “improved athletic performance” [Title/Abstract]) AND (“exercise-induced muscle damage” [Title/Abstract] OR “muscle soreness” [Title/Abstract] OR “muscle damage” [Title/Abstract]) AND (“eccentric exercise” [Title/Abstract]) AND (“inflammation” [Title/Abstract] OR “oxidative stress” [Title/Abstract]) AND (“benefits” [Title/Abstract]). In Title Abstract Keyword in All Text—with Publication Year from 2013 to 2024. Filters: Full text, Trial, in the last 10 years	51
**Web of Science**	((omega-3 OR omega-3 supplementation OR Polyunsaturated fatty acids (topic)) AND (“muscle recovery”), AND ((athletic performance OR improved athletic performance (topic)) AND ((exercise-induced muscle damage OR muscle soreness OR muscle damage(topic)) AND ((eccentric exercise (topic)) AND ((inflammation OR oxidative stress (topic)) AND ((benefits (topic)). Anywhere Publication 2013-2024, Filters: Full text, Trial, in the last 10 years	768

## References

[1] Fernández-Lázaro D., Mielgo-Ayuso J., Calvo J.S., Martínez A.C., García A.C., Fernandez-Lazaro C.I. (2020). Modulation of Exercise-Induced Muscle Damage, Inflammation, and Oxidative Markers by Curcumin Supplementation in a Physically Active Population: A Systematic Review. Nutrients.

[2] Gammone M.A., Riccioni G., Parrinello G., Orazio N.D. (2018). Omega-3 Polyunsaturated Fatty Acids: Benefits and Endpoints in Sport. Nutrients.

[3] Mittal M., Siddiqui M.R., Tran K., Reddy S.P., Malik A.B. (2014). Reactive Oxygen Species in Inflammation and Tissue Injury. Antioxid. Redox Signal..

[4] Biswas S.K. (2016). Does the Interdependence between Oxidative Stress and Inflammation Explain the Antioxidant Paradox?. Oxidative Med. Cell. Longev..

[5] Škrgat S., Korošec P., Kern I., Šilar M., Šelb J., Fležar M., Marčun R. (2018). Systemic and Airway Oxidative Stress in Competitive Swimmers. Respir. Med..

[6] Reid M.B. (2001). Nitric oxide, reactive oxygen species, and skeletal muscle contraction. Med. Sci. Sports Exerc..

[7] Miguel A.M.C.S., Roche E., Herranz-López M., Miguel M.C.S., Mielgo-Ayuso J., Fernández-Lázaro D. (2024). Impact of Melatonin Supplementation on Sports Performance and Circulating Biomarkers in Highly Trained Athletes: A Systematic Review of Randomized Controlled Trials. Nutrients.

[8] Mielgo-Ayuso J., Fernández-Lázaro D. (2021). Nutrition and Muscle Recovery. Nutrients.

[9] Calder P.C. (2018). Very long-chain n-3 fatty acids and human health: Fact, fiction, and the future. Proc. Nutr. Soc..

[10] Zaboli G., Igl W., Johansson A.C.V., Ameur A., Enroth S., Rivas M.A., Daly M.J., Schmitz G., Hicks A.A., Meitinger T. (2012). Genetic Adaptation of Fatty-Acid Metabolism: A Human-Specific Haplotype Increasing the Biosynthesis of Long-Chain Omega-3 and Omega-6 Fatty Acids. Am. J. Hum. Genet..

[11] Black K.E., Witard O.C., Baker D., Healey P., Lewis V., Tavares F., Christensen S., Pease T., Smith B. (2018). Adding Omega-3 Fatty Acids to a Protein-Based Supplement during Pre-Season Training Results in Reduced Muscle Soreness and the Better Maintenance of Explosive Power in Professional Rugby Union Players. Eur. J. Sport. Sci..

[12] Mickleborough T.D., Lindley M.R., Ionescu A.A., Fly A.D. (2006). Protective Effect of Fish Oil Supplementation on Exercise-Induced Bronchoconstriction in Asthma. Chest.

[13] Rees D., Miles E.A., Banerjee T., Wells S.J., Roynette C.E., Wahle K.W., Calder P.C. (2006). Dose-Related Effects of Eicosapentaenoic Acid on Innate Immune Function in Healthy Humans: A Comparison of Young and Older Men. Am. J. Clin. Nutr..

[14] Smith G.I., Julliand S., Reeds D.N., Sinacore D.R., Klein S., Mittendorfer B. (2015). Fish Oil-Derived n-3 PUFA Therapy Increases Muscle Mass and Function in Healthy Older Adults. Am. J. Clin. Nutr..

[15] Tinsley G.M., Gann J.J., Huber S.R., Andre T.L., La Bounty P.M., Bowden R.G., Gordon P.M., Grandjean P.W. (2017). Effects of Fish Oil Supplementation on Postresistance Exercise Muscle Soreness. J. Diet. Suppl..

[16] Lembke P., Capodice J., Hebert K., Swenson T. (2014). Influence of Omega-3 (N3) Index on Performance and Wellbeing in Young Adults after Heavy Eccentric Exercise. J. Sports Sci. Med..

[17] Tsuchiya Y., Yanagimoto K., Nakazato K., Hayamizu K., Ochi E. (2016). Eicosapentaenoic and Docosahexaenoic Acids-Rich Fish Oil Supplementation Attenuates Strength Loss and Limited Joint Range of Motion after Eccentric Contractions: A Randomized, Double-Blind, Placebo-Controlled, Parallel-Group Trial. Eur. J. Appl. Physiol..

[18] Camandola S., Leonarduzzi G., Musso T., Varesio L., Carini R., Scavazza A., Chiarpotto E., Baeuerle P.A., Poli G. (1996). Nuclear Factor KB Is Activated by Arachidonic Acid but Not by Eicosapentaenoic Acid. Biochem. Biophys. Res. Commun..

[19] Kumar A., Takada Y., Boriek A.M., Aggarwal B.B. (2004). Nuclear Factor-KappaB: Its Role in Health and Disease. J. Mol. Med..

[20] Ochi E., Tsuchiya Y., Yanagimoto K. (2017). Effect of eicosapentaenoic acids-rich fish oil supplementation on motor nerve function after eccentric contractions. J. Int. Soc. Sports Nutr..

[21] Tartibian B., Maleki B.H., Abbasi A. (2011). Omega-3 Fatty Acids Supplementation Attenuates Inflammatory Markers after Eccentric Exercise in Untrained Men. Clin. J. Sport Med..

[22] Gladman S.J., Huang W., Lim S.-N., Dyall S.C., Boddy S., Kang J.X., Knight M.M., Priestley J.V., Michael-Titus A.T. (2012). Improved Outcome after Peripheral Nerve Injury in Mice with Increased Levels of Endogenous ω-3 Polyunsaturated Fatty Acids. J. Neurosci. Off. J. Soc. Neurosci..

[23] Patten G.S., Abeywardena M.Y., McMurchie E.J., Jahangiri A. (2002). Dietary Fish Oil Increases Acetylcholine- and Eicosanoid-Induced Contractility of Isolated Rat Ileum. J. Nutr..

[24] Tartibian B., Maleki B.H., Abbasi A. (2009). The Effects of Ingestion of Omega-3 Fatty Acids on Perceived Pain and External Symptoms of Delayed Onset Muscle Soreness in Untrained Men. Clin. J. Sport Med..

[25] Biolo G., Tipton K.D., Klein S., Wolfe R.R. (1997). An Abundant Supply of Amino Acids Enhances the Metabolic Effect of Exercise on Muscle Protein. Am. J. Physiol..

[26] Mcglory C., Wardle S.L., Macnaughton L.S., Witard O.C., Scott F., Dick J., Bell J.G., Phillips S.M., Galloway S.D.R., Hamilton D.L. (2016). Fish oil supplementation suppresses resistance exercise and feeding-induced increases in anabolic signaling without affecting myofibrillar protein synthesis in young men. Physiol. Rep..

[27] Lewis E.J.H., Radonic P.W., Wolever T.M.S., Wells G.D., Lewis E.J.H., Radonic P.W., Wolever T.M.S., Lewis E.J.H., Radonic P.W., Wolever T.M.S. (2015). 21 days of mammalian omega-3 fatty acid supplementation improves aspects of neuromuscular function and performance in male athletes compared to olive oil placebo. J. Int. Soc. Sports Nutr..

[28] Ardern C.L., Büttner F., Andrade R., Weir A., Ashe M.C., Holden S., Impellizzeri F.M., Delahunt E., Dijkstra H.P., Mathieson S. (2022). Implementing the 27 PRISMA 2020 Statement items for systematic reviews in the sport and exercise medicine, musculoskeletal rehabilitation and sports science fields: The PERSiST (implementing Prisma in Exercise, Rehabilitation, Sport medicine and SporTs science) guidance. Br. J. Sports Med..

[29] da Costa Santos C.M., de Mattos Pimenta C.A., Nobre M.R.C. (2007). The PICO Strategy for the Research Question Construction and Evidence Search. Rev. Lat. Am. Enferm..

[30] Law M., Stewart D., Letts L., Pollock N., Bosch J., Westmorland M. (1998). Guidelines for Critical Review of Qualitative Studies.

[31] Moseley A.M., Elkins M.R., Van derWees P.J., Pinheiro M.B. (2020). Using research to guide practice: The Physiotherapy Evidence Database (PEDro). Braz. J. Phys. Ther..

[32] Higgins J.P.T., Eldridge S., Li T. (2021). Cochrane Handbook for Systematic Reviews of Interventions Version 6.2.

[33] Tomczyk M., Bidzan-Wiącek M., Kortas J.A., Kochanowicz M., Jost Z., Fisk H.L., Calder P.C., Antosiewicz J. (2024). Omega-3 fatty acid supplementation affects tryptophan metabolism during a 12-week endurance training in amateur runners: A randomized controlled trial. Sci. Rep..

[34] Tsuchiya Y., Ueda H., Yanagimoto K., Kato A., Ochi E. (2021). 4-Week Eicosapentaenoic Acid-Rich Fish Oil Supplementation Partially Protects Muscular Damage Following Eccentric Contractions. J. Int. Soc. Sports Nutr..

[35] Vandusseldorp T.A., Escobar K.A., Johnson K.E., Stratton M.T., Moriarty T., Kerksick C.M., Mangine G.T., Holmes A.J., Lee M., Endito M.R. (2020). Impact of Varying Dosages of Fish Oil on Recovery and Soreness Following Eccentric Exercise. Nutrients.

[36] Ávila-Gandía V., Torregrosa-García A., Luque-Rubia A.J., Abellán-Ruiz M.S., Victoria-Montesinos D., López-Román F.J. (2020). Re-esterified DHA improves ventilatory threshold 2 in competitive amateur cyclists. J. Int. Soc. Sports Nutr..

[37] Barquilha G., Miguel C., Dos M., Caçula K.G., Polotow T.G., Vasconcellos C.V., Fernandes A., Rodrigues L.E., Lambertucci R.H., Duarte T. (2023). Fish Oil Supplementation Improves the Repeated-Bout Effect Strength Training. Nutrients.

[38] Brook M.S., Din U., Tarum J., Selby A., Quinlan J., Bass J.J., Gharahdaghi N., Boereboom C., Abdulla H., Franchi M.V. (2021). Omega-3 supplementation during unilateral resistance exercise training in older women: A within subject and double-blind placebo-controlled trial. Clin. Nutr. ESPEN.

[39] Heileson J.L., Machek S.B., Harris D.R., Tomek S., de Souza L.C., Kieffer A.J., Barringer N.D., Gallucci A., Forsse J.S., Funderburk L.L.K. (2023). The effect of fish oil supplementation on resistance training-induced adaptations. J. Int. Soc. Sports Nutr..

[40] Jakeman J.R., Lambrick D.M., Wooley B., Babraj J.A., Faulkner J.A. (2017). Effect of an Acute Dose of Omega-3 Fish Oil Following Exercise-Induced Muscle Damage. Eur. J. Appl. Physiol..

[41] Lee S.-R., Directo D., Khamoui A. (2022). V Fish Oil Administration Combined with Resistance Exercise Training Improves Strength, Resting Metabolic Rate, and Inflammation in Older Adults. Aging Clin. Exp. Res..

[42] Mullins V.A., Graham S., Cummings D., Wood A., Ovando V., Skulas-Ray A.C., Polian D., Wang Y., Hernandez G.D., Lopez C.M. (2022). Effects of Fish Oil on Biomarkers of Axonal Injury and Inflammation in American Football Players: A Placebo-Controlled Randomized Controlled Trial. Nutrients.

[43] Nieman D.C., Gillitt N.D., Meaney M.P., Dew D.A. (2015). No Positive Influence of Ingesting Chia Seed Oil on Human Running Performance. Nutrients.

[44] Hou T.Y., McMurray D.N., Chapkin R.S. (2016). Omega-3 fatty acids, lipid rafts, and T cell signaling. Eur. J. Pharmacol..

[45] Ramos-Campo D.J., Ávila-Gandía V., López-Román F.J., Miñarro J., Contreras C., Soto-Méndez F., Domingo Pedrol J.C., Luque-Rubia A.J. (2020). Supplementation of Re-Esterified Docosahexaenoic and Eicosapentaenoic Acids Reduce Inflammatory and Muscle Damage Markers after Exercise in Endurance Athletes: A Randomized, Controlled Crossover Trial. Nutrients.

[46] Yang S., He Q., Shi L., Wu Y. (2023). Impact of Antarctic krill oil supplementation on skeletal muscle injury recovery after resistance exercise. Eur. J. Nutr..

[47] Urdampilleta A., López-Grueso R., Martínez-Sanz J.M., Mielgo-Ayuso J. (2014). Basic biochemical, hematological and hormonal parameters for monitoring the health and nutritional status in athletes. Rev. Esp. Nutr. Hum. Diet..

[48] Kyriakidou Y., Wood C., Ferrier C., Dolci A., Elliott B. (2021). The Effect of Omega-3 Polyunsaturated Fatty Acid Supplementation on Exercise-Induced Muscle Damage. J. Int. Soc. Sports Nutr..

[49] Lange K.W., Nakamura Y., Gosslau A.M., Li S. (2019). Are there serious adverse effects of omega-3 polyunsaturated fatty acid supplements?. J. Food Bioact..

[50] Jeromson S., Gallagher I.J., Galloway S.D.R., Hamilton D.L. (2015). Omega-3 Fatty Acids and Skeletal Muscle Health. Mar. Drugs.

[51] Tachtsis B., Camera D., Lacham-Kaplan O. (2018). Potential Roles of N-3 PUFAs during Skeletal Muscle Growth and Regeneration. Nutrients.

[52] DiLorenzo F.M., Drager C.J., Rankin J.W. (2014). Docosahexaenoic Acid Affects Markers of Inflammation and Muscle Damage after Eccentric Exercise. J. Strength Cond. Res..

[53] Calder P.C. (2017). Omega-3 Fatty Acids and Inflammatory Processes: From Molecules to Man. Biochem. Soc. Trans..

[54] Canals-Garzón C., Guisado-Barrilao R., Martínez-García D., Chirosa-Ríos I.J., Jerez-Mayorga D., Guisado-Requena I.M. (2022). Effect of Antioxidant Supplementation on Markers of Oxidative Stress and Muscle Damage after Strength Exercise: A Systematic Review. Int. J. Environ. Res. Public Health.

[55] Bloomer R.J., Larson D.E., Fisher-Wellman K.H., Galpin A.J., Schilling B.K. (2009). Effect of Eicosapentaenoic and Docosahexaenoic Acid on Resting and Exercise-Induced Inflammatory and Oxidative Stress Biomarkers: A Randomized, Placebo Controlled, Cross-over Study. Lipids Health Dis..

[56] Gray P., Chappell A., Jenkinson A.M., Thies F., Gray S.R. (2014). Fish Oil Supplementation Reduces Markers of Oxidative Stress but Not Muscle Soreness after Eccentric Exercise. Int. J. Sport. Nutr. Exerc. Metab..

[57] Da Boit M., Mastalurova I., Brazaite G., McGovern N., Thompson K., Gray S.R. (2015). The Effect of Krill Oil Supplementation on Exercise Performance and Markers of Immune Function. PLoS ONE.

[58] Pereira Panza V.S., Diefenthaeler F., da Silva E.L. (2015). Benefits of Dietary Phytochemical Supplementation on Eccentric Exercise-Induced Muscle Damage: Is Including Antioxidants Enough?. Nutrition.

[59] Philpott J.D., Donnelly C., Walshe I.H., MacKinley E.E., Dick J., Galloway S.D.R., Tipton K.D., Witard O.C. (2018). Adding Fish Oil to Whey Protein, Leucine, and Carbohydrate Over a Six-Week Supplementation Period Attenuates Muscle Soreness Following Eccentric Exercise in Competitive Soccer Players. Int. J. Sport. Nutr. Exerc. Metab..

[60] Chalchat E., Gaston A.-F., Charlot K., Peñailillo L., Valdés O., Tardo-Dino P.-E., Nosaka K., Martin V., Garcia-Vicencio S., Siracusa J. (2022). Appropriateness of Indirect Markers of Muscle Damage Following Lower Limbs Eccentric-Biased Exercises: A Systematic Review with Meta-Analysis. PLoS ONE.

[61] Lv Z., Zhang J., Zhu W. (2020). Omega-3 Polyunsaturated Fatty Acid Supplementation for Reducing Muscle Soreness after Eccentric Exercise: A Systematic Review and Meta-Analysis of Randomized Controlled Trials. Biomed. Res. Int..

[62] Mason S.A., Trewin A.J., Parker L., Wadley G.D. (2020). Antioxidant Supplements and Endurance Exercise: Current Evidence and Mechanistic Insights. Redox Biol..

[63] Fayh A.P.T., Borges K., Cunha G.S., Krause M., Rocha R., de Bittencourt P.I.H.J., Moreira J.C.F., Friedman R., da Silva Rossato J., Fernandes J.R. (2018). Effects of N-3 Fatty Acids and Exercise on Oxidative Stress Parameters in Type 2 Diabetic: A Randomized Clinical Trial. J. Int. Soc. Sports Nutr..

[64] Buonocore D., Verri M., Giolitto A., Doria E., Ghitti M., Dossena M. (2020). Effect of 8-Week n-3 Fatty-Acid Supplementation on Oxidative Stress and Inflammation in Middle- and Long-Distance Running Athletes: A Pilot Study. J. Int. Soc. Sports Nutr..

[65] Drobnic F., Storsve A.B., Burri L., Ding Y., Banquells M., Riera J., Björk P., Ferrer-Roca V., Domingo J.C. (2021). Krill-Oil-Dependent Increases in HS-Omega-3 Index, Plasma Choline and Antioxidant Capacity in Well-Conditioned Power Training Athletes. Nutrients.

[66] Berge R.K., Ramsvik M.S., Bohov P., Svardal A., Nordrehaug J.E., Rostrup E., Bruheim I., Bjørndal B. (2015). Krill Oil Reduces Plasma Triacylglycerol Level and Improves Related Lipoprotein Particle Concentration, Fatty Acid Composition and Redox Status in Healthy Young Adults—A Pilot Study. Lipids Health Dis..

[67] Marques C.G., Santos V.C., Levada-Pires A.C., Jacintho T.M., Gorjão R., Pithon-Curi T.C., Cury-Boaventura M.F. (2015). Effects of DHA-Rich Fish Oil Supplementation on the Lipid Profile, Markers of Muscle Damage, and Neutrophil Function in Wheelchair Basketball Athletes before and after Acute Exercise. Appl. Physiol. Nutr. Metab..

[68] Atashak S., Sharafi H., Azarbayjani M.A. (2013). Effect of Omega-3 Supplementation On The Blood Levels Of Oxidative Stress. Muscle Damage Inflamm. Markers After Acute Resist..

[69] De Salazar L., Contreras C., Torregrosa-García A., Luque-Rubia A.J., Ávila-Gandía V., Domingo J.C., López-Román F.J. (2020). Oxidative Stress in Endurance Cycling Is Reduced Dose-Dependently after One Month of Re-Esterified DHA Supplementation. Antioxidants.

[70] McKinley-Barnard S.K., Andre T.L., Gann J.J., Hwang P.S., Willoughby D.S. (2018). Effectiveness of Fish Oil Supplementation in Attenuating Exercise-Induced Muscle Damage in Women During Midfollicular and Midluteal Menstrual Phases. J. Strength Cond. Res..

[71] Luostarinen R., Saldeen T. (1996). Dietary Fish Oil Decreases Superoxide Generation by Human Neutrophils: Relation to Cyclooxygenase Pathway and Lysosomal Enzyme Release. Prostaglandins Leukot. Essent. Fatty Acids.

[72] Fisher M., Levine P.H., Weiner B.H., Johnson M.H., Doyle E.M., Ellis P.A., Hoogasian J.J. (1990). Dietary N-3 Fatty Acid Supplementation Reduces Superoxide Production and Chemiluminescence in a Monocyte-Enriched Preparation of Leukocytes. Am. J. Clin. Nutr..

[73] Lenn J., Uhl T., Mattacola C., Boissonneault G., Yates J., Ibrahim W., Bruckner G. (2002). The Effects of Fish Oil and Isoflavones on Delayed Onset Muscle Soreness. Med. Sci. Sports Exerc..

[74] Owen J.B., Butterfield D.A. (2010). Measurement of Oxidized/Reduced Glutathione Ratio. Methods Mol. Biol..

[75] Owens D., Twist C., Cobley J., Howatson G., Close G. (2019). Exercise-induced muscle damage: What is it, what causes it and what are the nutritional solutions?. Eur. J. Sport Sci..

[76] Ochi E., Tsuchiya Y. (2018). Eicosapentaenoic Acid (EPA) and Docosahexaenoic Acid (DHA) in Muscle Damage and Function. Nutrients.

[77] Ross B.M. (2008). The emerging role of eicosapentaenoic acid as an important psychoactive natural product: Some answers but a lot more questions. Lipid Insights.

[78] Tipton K.D. (2015). Nutritional Support for Exercise-Induced Injuries. Sport. Med..

[79] Fernández-Lázaro D., Mielgo-Ayuso J., Del Valle Soto M., Adams D.P., Gutiérrez-Abejón E., Seco-Calvo J. (2021). Impact of Optimal Timing of Intake of Multi-Ingredient Performance Supplements on Sports Performance, Muscular Damage, and Hormonal Behavior across a Ten-Week Training Camp in Elite Cyclists: A Randomized Clinical Trial. Nutrients.

[80] Fernández-Lázaro D., Garrosa E., Seco-Calvo J., Garrosa M. (2022). Potential Satellite Cell-Linked Biomarkers in Aging Skeletal Muscle Tissue: Proteomics and Proteogenomics to Monitor Sarcopenia. Proteomes.

